# Competition between skin antimicrobial peptides and commensal bacteria in type 2 inflammation enables survival of *S. aureus*

**DOI:** 10.1016/j.celrep.2023.112494

**Published:** 2023-05-10

**Authors:** Teruaki Nakatsuji, Samantha L. Brinton, Kellen J. Cavagnero, Alan M. O’Neill, Yang Chen, Tatsuya Dokoshi, Anna M. Butcher, Olive C. Osuoji, Faiza Shafiq, Josh L. Espinoza, Christopher L. Dupont, Tissa R. Hata, Richard L. Gallo

**Affiliations:** 1 Department of Dermatology, University of California, San Diego, La Jolla, CA 92037, USA; 2 Genomic Medicine, J. Craig Venter Institute, La Jolla, CA 92037, USA; 3 Lead contact

## Abstract

During inflammation, the skin deploys antimicrobial peptides (AMPs) yet during allergic inflammation it becomes more susceptible to *Staphylococcus aureus*. To understand this contradiction, single-cell sequencing of *Il4ra*^−/−^ mice combined with skin microbiome analysis reveals that lower production of AMPs from interleukin-4 receptor α (IL-4Rα) activation selectively inhibits survival of antibiotic-producing strains of coagulase-negative *Staphylococcus* (CoNS). Diminished AMPs under conditions of T helper type 2 (Th2) inflammation enable expansion of CoNS strains without antibiotic activity and increase *Staphylococcus aureus* (*S. aureus*), recapitulating the microbiome on humans with atopic dermatitis. This response is rescued in *Camp*^−/−^ mice or after topical steroids, since further inhibition of AMPs enables survival of antibiotic-producing CoNS strains. In conditions of Th17 inflammation, a higher expression of host AMPs is sufficient to directly inhibit *S. aureus* survival. These results show that antimicrobials produced by the host and commensal bacteria each act to control *S. aureus* on the skin.

## In brief

Nakatsuji et al. report how activation of IL-4Rα partially suppresses production of antimicrobial peptides from the skin so that they cannot kill *Staphylococcus aureus* (*S. aureus*) but do inhibit protective strains of *Staphylococcus* that produce bacteriocins. Their findings can explain why *S. aureus* expands on the skin of patients with atopic dermatitis.

## Graphical Abstract

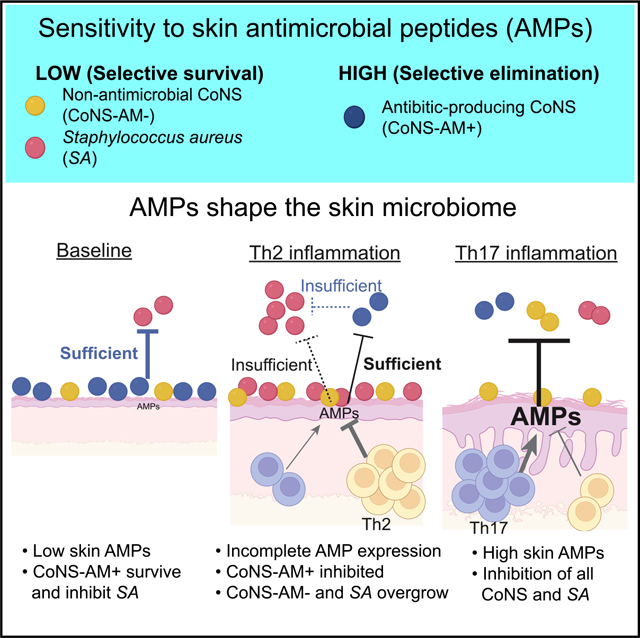

## INTRODUCTION

Skin in allergic skin diseases with T helper type 2 (Th2) inflammation, such as atopic dermatitis, is more susceptible to skin infections and has a greater absolute abundance of *Staphylococcus aureus* than healthy skin. This overgrowth of *Staphylococcus aureus* (*S. aureus*) is different than that found on patients with psoriasis, a form of Th17-dominant inflammation, where the relative proportion of *S. aureus* can be increased, but the absolute abundance of *Staphylococcus* is similar to healthy skin and does not promote increased infections.^1,2^ Despite the major health impact of infections in Th2 inflammatory disorders, it is not understood why *S. aureus* overpopulates type 2 forms of skin inflammation.

The production of antimicrobial peptides (AMPs) by the skin and other epithelial surfaces is an essential system for defense against invasion by *S. aureus*. AMPs are either induced or expressed constitutively by many cell types, including keratinocytes, immune cells, sebocytes, and dermal preadipocytes.^3^ Regulation of this system appears important for maintaining health, as lack of appropriate AMP production closely associates with increased susceptibility to infections,^4–7^ while overproduction of some AMPs can drive pathogenic inflammation due to pro-inflammatory functions that are inherent in many of the AMPs.^8,9^

The expression of AMPs such as cathelicidin and some β-defensins is partially inhibited on lesional skin of patients with atopic dermatitis and is much less than that observed on Th17-dominant inflammatory responses such as wounding or psoriasis.^10^ The suppression of these AMPs occurs in part through the action of interleukin-4 (IL-4) and IL-13.^11,12^ An over-growth of other species of *Staphylococci*, in particular *S. epidermidis*,^2,13,14^ is also seen in Th2 inflammation. Increased skin colonization by *S. epidermidis* or *S. aureus* and their production of a variety of superantigens, proteases, and toxins promote further exacerbation of disease, causing immune dysregulation, mast cell degranulation, and barrier disruption.^15,16^ Understanding why these characteristic changes in the skin microbiome occur is an important question that remains unanswered.

In this study, we sought to understand factors that enable growth of *S. aureus* on the skin during Th2 inflammation. We co-ordinated analysis of host defense functions with analysis of the skin commensal bacteria that could act to benefit the skin. These responses were compared in murine models of Th2 inflammation without function of the IL-4 receptor α (*Il4rα* ^−/−^*)* or mice lacking the capacity to express the host AMP cathelicidin (*Camp*^−/−^). The composition of the skin microbiome was then further compared during anti-inflammatory therapy and in models of Th17 inflammation or on non-inflamed skin. This analysis reveals how cooperation between both host AMPs and antibiotics from commensal microbes is required to resist *S. aureus* and shape the microbial community, demonstrating that both AMPs and commensal bacteria are required for defense of the skin.

## RESULTS

### Activation of the IL-4Rα suppresses skin defense against *S. aureus*

To investigate the interaction between Th2 inflammation and *S. aureus* colonization, we applied two different murine models, topical MC903 or sensitization by ovalbumin (OVA).^17,18^ We em ployed alternate Th2 models since OVA sensitization initiates maximal Th2 inflammation only on mice with a filaggrin gene mu tation (*Flg^ft/ft^*), while MC903 is an alternative but rapid and direct approach to achieve a similar Th2-skewed response that could be adapted to other mouse genetic systems. In the presence or absence of *S. aureus*, both models were confirmed to induce a Th2 inflammatory response, including an increase in *Il4*, *Il13*, *Tslp*, and *Il17a* and an increase in *Camp* and *Defb14* relative to the baseline (Figures S1A–S1F and S1H–S1L). In contrast, without OVA or MC903 pretreatment, *S. aureus* application resulted in a more predominant Th17 response with a greater increase in AMPs. *S. aureus* survival increased on the skin after pretreatment with MC903 or OVA relative to untreated mice (Figures S1G and S1M) and *S. aureus* further promoted the *Il4* expression (Figures S1B and S1I). These data validated that initiation of Th2 inflammation by MC903 or OVA alters the immune response to *S. aureus* and the capacity of *S. aureus* to colonize the skin.

**Figure 1.**
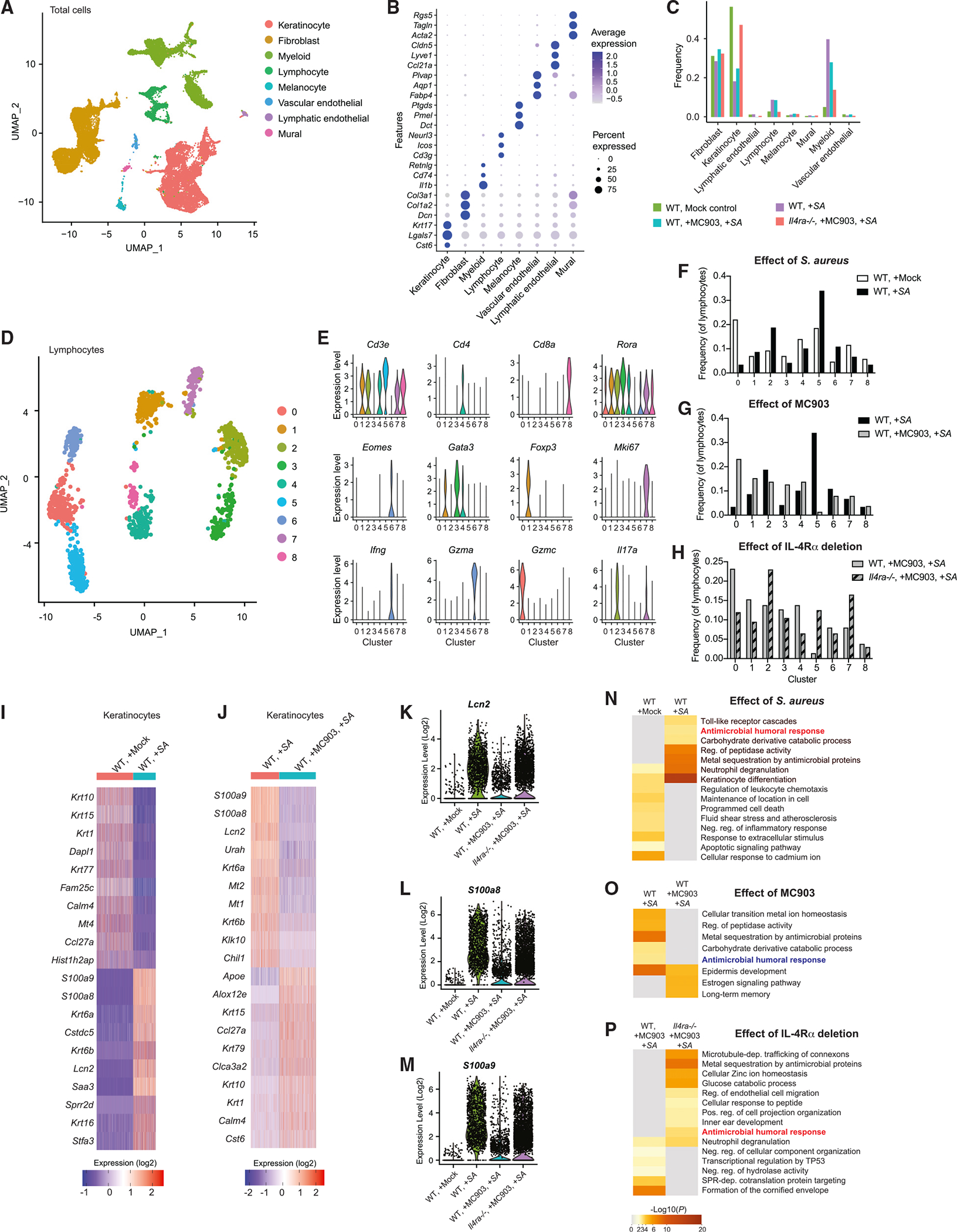
Th2 signaling suppresses host defense of the skin against *S. aureus* (A and B) Cell clusters identified by UMAP plot (A) based on expression of transcription markers (B) in non-inflamed skin of WT mice, inflamed skin of WT mice treated with *S. aureus*, WT mice treated with MC903 and *S. aureus*, or *Il4ra*^−/−^ mice treated with MC903 and *S. aureus*. (C) Frequency of the 8 major cell populations in the skin from mice treated as in (A) and (B).(C) UMAP plot of lymphocyte populations in the skin from mice treated as in (A) and (B). (D) Violin plots of conserved lymphocyte marker gene expression in lymphocyte clusters. (F–H) Comparison of relative abundance of lymphocyte clusters identified by UMAP plot. (I and J) Heatmap of top 10 genes upregulated and downregulated in total keratinocyte populations by *S. aureus* application in WT mice (I) or after treatment of MC903 in WT mice that received *S. aureus* application (J). (K–M) Gene expression of antimicrobial host defense genes, *Lcn2* (K), *S100a8* (L), and *S100a9* (M), in keratinocyte populations from each indicated mouse group treated as in (A) and (B). (N–P) Heatmap of selected Gene Ontogeny terms upregulated and downregulated in total keratinocyte populations by *S. aureus* application in WT mice (N), in mice treated with MC903 (O), and in the absence of IL-4Ra in mice treated by MC903 and *S. aureus* (P). All data in this figure were obtained from 10,000 live cell suspensions pooled equally from five 8 mm punch biopsies of mice independently treated in each group (n = 5). Data were obtained from a single scRNA-seq run.

With validation that Th2 inflammation enabled *S. aureus* survival, we next analyzed the response of *Il4ra*^−/−^ mice by single-cell RNA sequencing (scRNA-seq) to more broadly examine the changes in cell-specific host defense that occur in the skin due to activation of the IL-4 receptor. Uniform manifold approximation and projection (UMAP) analysis from 27,023 cells that passed quality control (Figures S2A–S2D) defined 8 cell clusters with unique gene expression profiles (Figures 1A and 1B). Treatment of wild-type (WT) mice with *S. aureus* alone or MC903 followed by *S. aureus* resulted in a decrease in the cell frequency of keratinocytes compared with WT mice with mock control treatment (Figure 1C). In contrast, the frequency in *Il4ra*^−/−^ mice treated by MC903 followed by *S. aureus* increased compared with that in WT mice with mock treatment. Overall, several populations of cells were influenced by the deletion of *Il4ra* in a setting of Th2 inflammation and by *S. aureus* exposure (Figure 1C). In the lymphocyte population, 9 distinct lymphocyte clusters were identified (Figures 1D, 1E, and S2E). Topical application of *S. aureus* to WT mice increased the frequency of clusters 2 and 5, with cluster 2 expressing *Cd3e* and *Il17a* (Figures 1E and 1F). These observations were consistent with the expected dominant Th17 response to bacterial invasion^19^ and confirmed by qPCR (Figure S1A). In contrast, in control WT mice, when Th2 inflammation was induced by MC903 followed by *S. aureus*, increases in *Gata3*-expressing populations including *Cd3e*^+^ *Gata3^+^* Th2 cells in cluster 4, *Cd3e*^−^ *Gata3^+^* type 2 innate lymphoid cells (ILC2s) in cluster 3, and *Cd3e*^+^ *FoxP3^+^* regulatory T cells (Tregs) in cluster 1 were observed relative to *S. aureus* application without MC903 (Figures 1E and 1G). *Il4ra*^−/−^ reversed this response and shifted back toward an increased frequency of an *Il17a*^+^ subset (cluster 2) and a decrease in the frequency of the Th2 and ILC2 subsets (clusters 4 and 3, respectively) (Figures 1E and 1H). Similarly, natural killer cells identified in cluster 0 increased by MC903 and decreased in *Il4ra*^−/−^ mice under Th2 immune response, but their roles in Th2 response have been still largely unknown. Thus, signaling through the IL-4Rα was required to regulate equilibrium between Th17 and Th2 responses to MC903 and *S. aureus*. Since Th17 subsets enhance epidermal antimicrobial defense,^20^ these data validated the use of this model to further investigate the cutaneous antimicrobial milieu during conditions of Th2 inflammation.

Since the epidermis is the initial host barrier to *S. aureus* colonization, we next concentrated on examination of global changes in gene expression within the epidermal keratinocyte population. Exposure to *S. aureus* alone notably increased expression of several host defense genes in keratinocytes including *S100a8*, *S100a9*, and *Lcn2* in WT mice (Figure 1I). The host defense response to *S. aureus* was diminished when Th2 inflammation was initiated by MC903 prior to bacterial application in WT mice (Figure 1J). *Il4ra*^−/−^ skin did not show the diminished expression levels of these AMPs after treatment by MC903 and *S. aureus* (Figures 1K–1M). Gene Ontogeny analysis showed enrichment of genes related to “antimicrobial humoral response” upon exposure to *S. aureus* alone that was diminished when Th2 inflammation was initiated by MC903 in WT mice and restored in *Il4ra*^−/−^ mice with similar treatment (Figures 1N–1P). Thus, the keratinocyte host defense response was increased relative to non-inflamed skin by epicutaneous exposure to *S. aureus*, but IL-4Rα signaling suppressed the magnitude of this broad host defense response to *S. aureus*.

**Figure 2.**
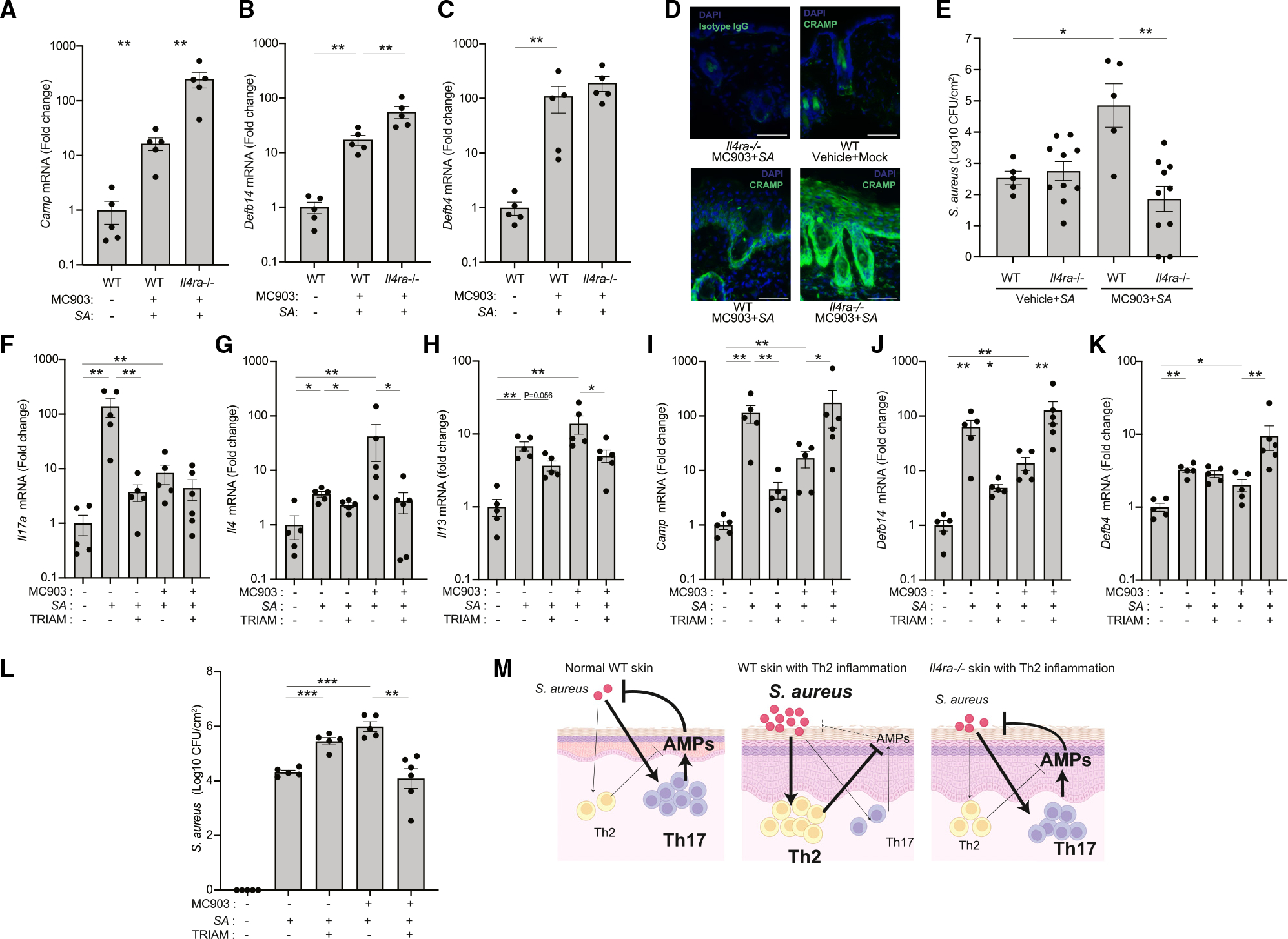
Absence of Th2 signaling enhances antimicrobial defense against *S. aureus* (A–C) Expression of *Camp* (A), *Defb14* (B), and *Defb4* (C) in the skin of WT or *Il4ra*^−/−^ mice after treatment with MC903 and *S. aureus* (*SA*) application. Each dot represents the average of duplicate technical replicates from an individual animal. Data represent mean ± SEM of 5 independent mice. p value (**p < 0.01) was calculated by two-tailed Mann-Whitney U test.n. (D) Immunofluorescence for CRAMP (a gene product of *Camp*) in the skin of WT or *Il4ra*^−/−^ mice after treatment with MC903 and *S. aureus* application. Scale bar: 100 μm. Image is representative of similar results from indicated 5 biological replicates. (E) Survival of *S. aureus* on the skin of WT or *Il4ra*^−/−^ mice pretreated with MC903 or vehicle for 48 h (WT: n = 5; *Il4ra*^−/−^: n = 10). Each dot represents the average of duplicate technical replicates from an individual animal. Data represent mean ± SEM of 5 (WT) or 10 (*Il4ra*^−/−^) independent mice. p value (*p < 0.05 and **p < 0.01) was calculated by two-tailed unpaired parametric t test. (F–L) Triamcinolone effects on the expression of *Il17a* (F), *Il4* (G), *Il13* (H), and AMP genes (I–K) and survival of *S. aureus* for 48 h in WT mice pretreated with MC903 or vehicle (L). Each dot represents the average of duplicate technical replicates from an individual animal. Data represent mean ± SEM of 5–6 biological replicates from independent mice as individually shown. p value (*p < 0.05, **p < 0.01, and ***p < 0.001) was calculated by two-tailed Mann-Whitney U test (F–K) or two-tailed unpaired parametric t test (L). (M) Illustration of the host antimicrobial peptide response to *S. aureus* colonization in presence or absence of Th2 inflammation and in WT or *Il4ra*^−/−^ skin.

We next sought to evaluate the expression of additional AMPs not easily detected by scRNA-seq that also influence the survival of *S. aureus*,^21,22^ including *Camp*, *Defb4*, and *Defb14.* Each of these AMPs increased in the skin after Th2 inflammation induced by MC903 followed by *S. aureus* but increased further under these conditions in *Il4ra*^−/−^ mice (Figures 2A–2C). Induction of Th2 inflammation with MC903 also suppressed the AMP response to skin wounding (Figure S3). Consistent with the expression of mRNA, protein expression of Cramp, the mature *Camp* gene product in mice, was also observed to slightly increase after treatment with MC903 and *S. aureus* but increased further in *Il4ra*^−/−^ mice (Figure 2D). The enhanced AMP response correlated with a decrease in *S. aureus* colonization observed in *Il4ra*^−/−^ mice during Th2 inflammation (Figure 2E). Overall, these findings show that while some increase in antimicrobial host defense is observed in the setting of Th2 inflammation alone, these responses to *S. aureus* are even greater in the absence of IL-4Rα signaling and result in improved defense against *S. aureus*.

To further understand the interactions between Th2 inflammation and host defense, we next evaluated the effects of topical application of a broad-acting anti-inflammatory steroid cream (triamcinolone). On healthy skin, triamcinolone led to decreased *Il17a*, *Camp*, and *Defb14* expression after *S. aureus* exposure (Figures 2F–2J). However, the opposite trend was observed in skin with Th2 inflammation induced by the combination of MC903 and *S. aureus*: under these conditions, topical triamcinolone increased expression of *Camp*, *Defb14*, and *Defb4* (Figures 2I–2K). The increase in AMP expression correlated with decreased *Il4* and *Il13* (Figures 2G and 2H) and decreased survival of *S. aureus* (Figure 2L). Taken together, Th2-inflamed skin treated with triamcinolone recapitulated the observations in *Il4ra*^−/−^ mice by showing that decreased expression of Th2 cytokines was associated with an increased host AMP response to *S. aureus* and decreased *S. aureus* colonization. Combined, our findings show that topical *S. aureus* colonization promotes a prominent AMP response in healthy skin, but in the setting of prior Th2 inflammation, AMPs are only partially increased, and this is associated with enhanced *S. aureus* survival (Figure 2M).

### Skin inflammation correlates to loss of CoNS strains that produce antimicrobial activity against *S. aureus* in human

**Figure 3.**
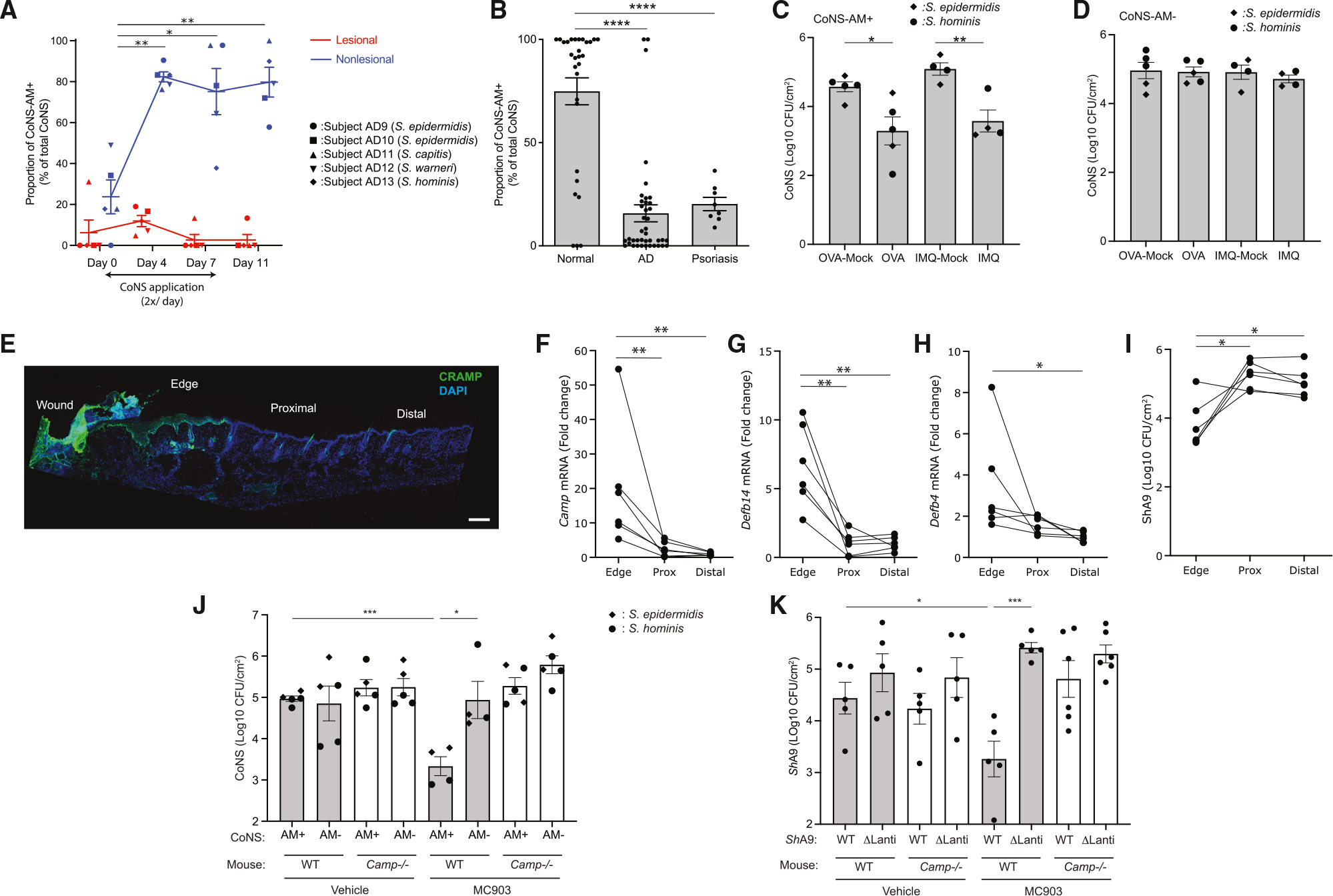
Th2 and Th17 inflammation inhibits survival of antibiotic-producing CoNS in human and mice (A) Survival of autologously derived antibiotic-producing CoNS strains (CoNS-AM+) of *S. epidermidis*, *S. capitis*, *S. warneri*, and *S. hominis* species on non-lesional and lesional skin of forearms of donor subjects with atopic dermatitis (AD). Bacteria were applied twice a day for 7 days. To estimate the survival of antimicrobial CoNS, the relative proportion of CoNS isolates with the capacity to inhibit *S. aureus* were measured at the time points indicated. On days 4 and 7, swab samples were obtained 4 h after the last application. Each dot represents data from an individual subject. Data represent mean ± SEM of 5 individuals who received indicated species of CoNS-AM+ (demographic data shown in Table S1). p value (*p < 0.05 and **p < 0.01) was calculated by two-tailed paired parametric t test to baseline data. (B) Frequency of CoNS-AM+ isolates on skin of control volunteers compared with inflamed skin of human subjects with AD or psoriasis. Each dot represents data from an individual subject. Data represent mean ± SEM (healthy: n = 29; AD: n = 40; psoriasis: n = 8). p value (****p < 0.0001) was calculated by two-tailed unpaired parametric t test. Demographic data shown in Table S2. (C and D) Survival of representative CoNS-AM+ or CoNS strains that do not produce antibiotics (CoNS-AM−) of *S. epidermidis* or *S. hominis* on OVA-sensitized or imiquimod-treated skin of *Flg*^*ft/ft*^ Balb/c mice after 48 h. Each dot represents the average of duplicate technical replicates from an individual animal. Data represent mean ± SEM of 4–5 independent mice treated by distinct bacterial strain. p value (*p < 0.05 and **p < 0.01) was calculated by two-tailed unpaired parametric t test. Antimicrobial activity of each CoNS strain is shown in Figure S5. (E) Immunostaining for CRAMP in the back skin of mice after partial thickness wounding. Scale bar: 400 mm. Image is representative of similar results from 6 biological replicates. (F–I) Expression of AMP genes (F–H) and survival of ShA9 (I) at the edge, proximal (Prox), and distal regions of the skin wound. Each dot represents the average of duplicate technical replicates from an individual animal (n = 6). p value (*p < 0.05 and **p < 0.01) was calculated by two-tailed paired parametric t test. (J and K) Survival of representative CoNS-AM+ or CoNS-AM− strains of *S. epidermidis* or *S. hominis* (J) or ShA9-WT or ShA9-ΔLanti (K) on MC903- or vehicle-treated skin of WT or *Camp*^−/−^ Balb/c mice 48 h after application. Each dot represents data from an individual animal. Data represent mean ± SEM of an independent animal that received distinct bacterial strains (J: n = 4 or 5) or that received WT or ΔLanti-ShA9 (K: n = 5 or 6). p value (*p < 0.05 and ***p < 0.001) was calculated by two-tailed unpaired parametric t test. Data in (J) represent survival of each CoNS strain on individual mouse. Each dot represents the average of duplicated technical replicates from an individual animal.

An increase in host antimicrobials during Th2 inflammation, even if less than the increase that occurs in healthy skin, could not pro mote survival of *S. aureus*. Either an absolute loss in a skin de fense mechanism or an improvement of the growth-promoting environment must take place. To examine if a loss in skin de fense may occur by depletion of skin protection by commensal bacteria, we next evaluated the survival of commensal coagu lase-negative *Staphylococcus* (CoNS) strains on the skin that can also kill *S. aureus*.^23^ A clinical trial was performed of anti biotic-producing CoNS strains that were autologously derived from subjects with atopic dermatitis and applied twice a day for 7 days to inflamed or non-inflamed skin of the autologous donor subject (Table S1).^24^ These antimicrobial-producing CoNS strains were from 4 different species: *S. epidermidis*, *S. capitis*, *S. warneri*, and *S. hominis*. Survival of these antimicro bial CoNS strains was evaluated by measuring the proportion of CoNS isolates with the capacity to inhibit *S. aureus* at different time points after application to the subject. CoNS with antimicro bial activity did not colonize as abundantly on inflamed skin compared with non-inflamed skin (Figure 3A). We also evaluated the colonization of antimicrobial CoNS strains on the inflamed skin of subjects with psoriasis, a Th17-dominant inflammatory skin disorder (Table S2). The subjects with psoriasis, like atopic dermatitis, also had a lower frequency of antibiotic-producing CoNS than on healthy skin (Figure 3B). Therefore, both Th2 and Th17 forms of inflammation correlated with decreased colo nization by antibiotic-producing CoNS. These observations show that inflammation selectively promotes the elimination of antibiotic-producing CoNS strains from human skin.

A direct comparison of the bacterial communities on the skin of subjects with atopic dermatitis or psoriasis by full-length 16S sequencing and qPCR confirmed prior reports^14,25^ that atopic dermatitis has a higher absolute abundance of total bacterial DNA as well as a higher proportion of *S. aureus* than psoriasis (Figure S4A). Subjects with psoriasis, although with lesser total bacterial abundance than atopic dermatitis, had an increase in the relative abundance of *S. aureus* compared with healthy controls (Figures S4B–S4D). Thus, neither skin barrier disruption from inflammation nor the colonization by CoNS with antimicrobial activity was sufficient to explain why an increase in *S. aureus* colonization occurs during Th2 inflammation.

### Expression of host AMPs inhibits survival of antibiotic-producing CoNS

To explore how different host defense responses may influence colonization of CoNS strains with antibiotic activity, we next applied representative strains of *S. epidermidis* or *S. hominis* with antimicrobial activity (Figure S5) to the skin of *Flg^ft/ft^* mice sensitized with OVA or mice treated with imiquimod. Similar to MC903, sensitization with OVA also effectively induced a *Il4-*dominant response, a partial increase of host AMPs, and an increased colonization by *S. aureus* (Figures S1J–S1M). In contrast, imiquimod application, which has been used as a model of psoriasis,^26^ induced a more *Il17a-*dominant response, higher levels of AMPs, and enhanced protection against *S. aureus* (Figure S6). These mouse models recapitulated the observations made of human subjects, as the survival of antibiotic-producing strains of CoNS decreased in both Th2- and Th17-inflammatory models compared with matched control skin that was not inflamed (Figure 3C). In contrast, strains of matched species that did not have antibiotic activity survived equally well on non-inflamed and inflamed skin (Figure 3D). The selective reduction of antibiotic-producing CoNS was also confirmed on an alternative model of acute inflammation induced by a partial-thickness skin wound, which results in a high induction of host AMPs.^27^ Immunohistochemical staining for the cathelicidin AMP and qPCR for *Camp*, *Defb4*, and *Defb14* showed a gradient of AMP expression from the highest at wound edge to the lowest at a distal site (Figures 3E–3H). Application of an antibiotic-producing CoNS strain (ShA9) across the entire skin showed that this organism was most inhibited at the site of highest AMP expression at the wound edge (Figure 3I). Thus, three different mouse models showed that antibiotic-producing CoNS strains have decreased viability on inflamed skin.

To further understand the mechanism responsible for the selective suppression of antibiotic-producing CoNS during inflammation, the survival of representative strains (Figure S5) of *S. epidermidis* or *S. hominis* with or without capacity to produce antimicrobial activity was evaluated on the back skin of *Camp*^−/−^ or WT-control mice treated with MC903 or vehicle (Figure 3J). All strains equally colonized the vehicle-treated non-inflamed skin of both genotypes. In contrast, after Th2 inflammation was induced, control mice selectively decreased the survival of anti biotic-producing CoNS strains but *Camp*^−/−^ mice did not.

To further evaluate if the capacity to produce antimicrobial activity correlated with strain survival in mice expressing *Camp*, lantibiotic genes in a strain of antibiotic-producing CoNS were deleted (ShA9-ΔLanti) and tested on *Camp*^−/−^ mice. This strain was previously confirmed to lack detectable antibiotic activity.^28^ ShA9-ΔLanti was not inhibited in any of the inflammatory mouse models tested (MC903, OVA, imiquimod), but the parental strain was inhibited (Figures 3K, S7A, and S7B). Conversely, inflamed skin of *Camp*^−/−^ mice did not suppress either parental or ShA9-ΔLanti strains (Figure 3K). This suggested that the production of lantibiotics by the parental ShA9 CoNS strain conferred sensitivity to AMPs and that the host expression of *Camp* was important to elimination of antibiotic-producing CoNS.

**Figure 4.**
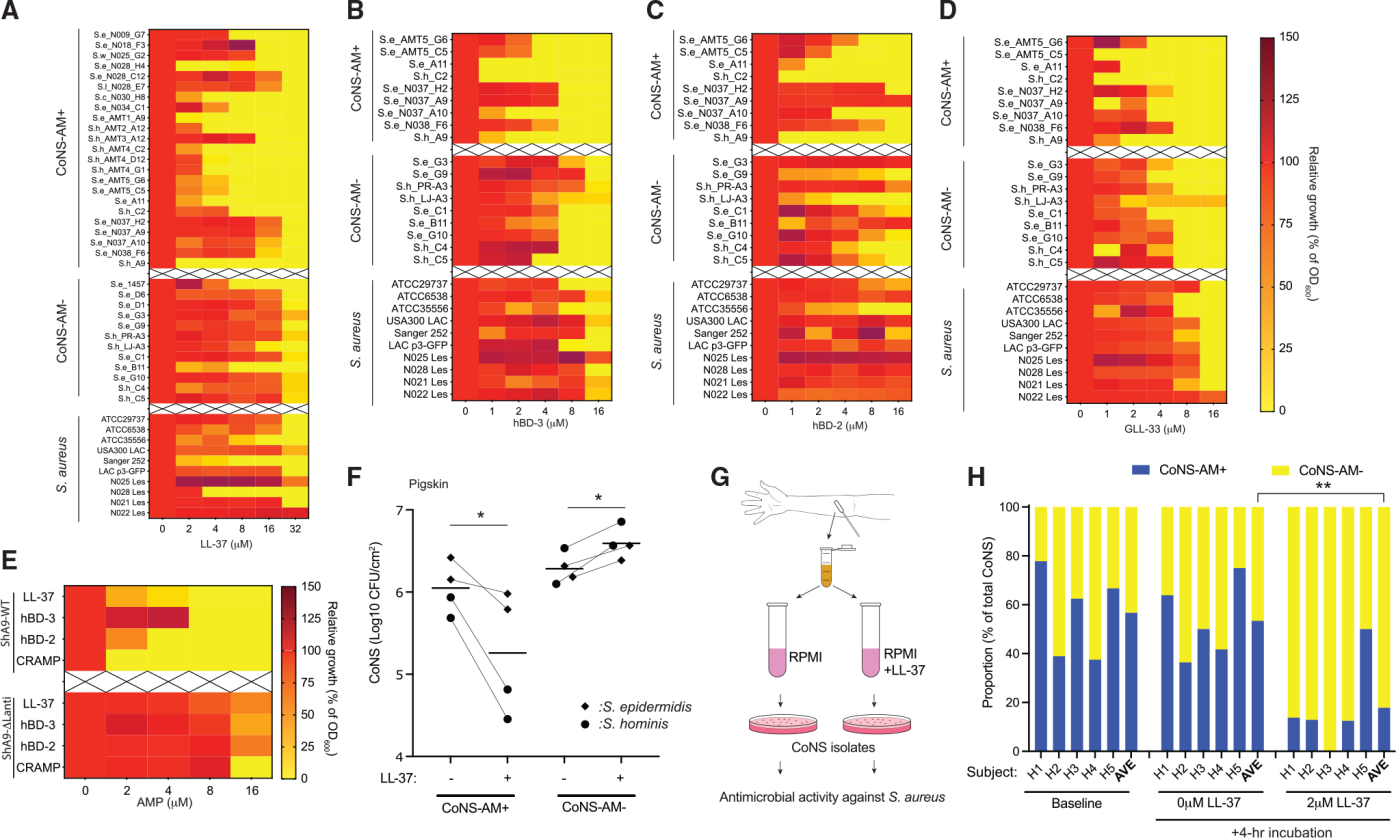
*S. aureus* and CoNS that do not produce antibiotic activity are more resistant to AMPs (A–D) Dose-dependent growth inhibition of representative strains of antimicrobial CoNS (CoNS-AM+), non-antimicrobial CoNS (CoNS-AM−), and *S. aureus* by human cathelicidin LL-37 (A), hBD-3 (B), hBD-2 (C), and mouse cathelicidin GLL-33 (D). Se, *S. epidermidis*; Sh, *S. hominis*; Sl, *S. lugdunensis*; Sc, *S. capitis*; Sw, *S. warneri*. Data represent mean of duplicate technical replicates. (E) Comparison of growth inhibition of ShA9-WT and a ShA9-ΔLanti by human and murine AMPs. Growth of bacteria was monitored by OD_600_, and scale of relative growth was shown by heatmap (A–E). Data represent the mean of duplicate technical replicates. (F) Effects of LL-37 on colonization by representative CoNS-AM+ or CoNS-AM− strains of *S. epidermidis* or *S. hominis* on pigskin sheet for 24 h. Each dot represents the average of duplicate technical replicates from an individual bacterial strain, and the horizontal bar represents the mean of 4 distinct bacterial strains. p value (*p < 0.05) was calculated by two-tailed unpaired parametric t test. (G and H) Effect of a low dose of LL-37 on the proportion of CoNS-AM+ and CoNS-AM− within the microbial community on the forearm skin of healthy human subjects *in vitro*. p value (*p < 0.05, **p < 0.01, and ***p < 0.001) was calculated by two-tailed paired parametric t test.

To explore if the changes in sensitivity to host AMPs by CoNS also could be seen with other CoNS species and AMPs, we next evaluated the growth *in vitro* of a library of *Staphylococcal* species and strains after culture with several different synthetic AMPs. Human cathelicidin (LL-37), β-defensins (hBD2 and hBD3), and the mouse cathelicidin (GLL-33) were each independently tested. Antimicrobial activity of the CoNS strains tested have been characterized previously.^23,24^ Most antibiotic-producing CoNS strains were more sensitive to inhibition by AMPs than CoNS strains without antibiotic activity, and *S. aureus* strains were also typically more resistant to AMPs (Figures 4A–4D and S8). Similarly, ShA9-WT was more sensitive to LL-37, hBD-3, hBD-2, and GLL-33 than ShA9-ΔLanti (Figure 4E).

Survival of CoNS on whole skin was also evaluated in strains of *S. epidermidis* or *S. hominis* that were applied *ex situ* to a sheet of pigskin, to which a physiological amount of LL-37 (2.5 nmol) was applied. This concentration approximated the amount of LL-37 observed on atopic lesional skin.^10^ Pigskin with LL-37 inhibited survival of strains of both species with antibiotic activity but did not inhibit strains without antibiotic activity (Figure 4F).

Finally, the capacity of AMPs to shape the composition of the skin CoNS community was evaluated by examining the effects of LL-37 on survival of mixed communities of bacteria obtained from human skin (Figure 4G). Skin swab samples were obtained from the forearms of 5 healthy subjects (Table S4), and these mixed bacterial samples were incubated in media without or with a low dose of LL-37 for 4 h. These populations did not have *S. aureus*. LL-37 decreased the relative proportion of CoNS with antimicrobial activity but incubation without LL-37 did not (Figure 4H). Taken together, these observations demonstrate that host AMPs selectively inhibit the survival of CoNS strains that express antibiotic activity against *S. aureus*.

### Synergy between mammalian AMPs and lantibiotics is self-destructive to antibiotic-producing CoNS

**Figure 5.**
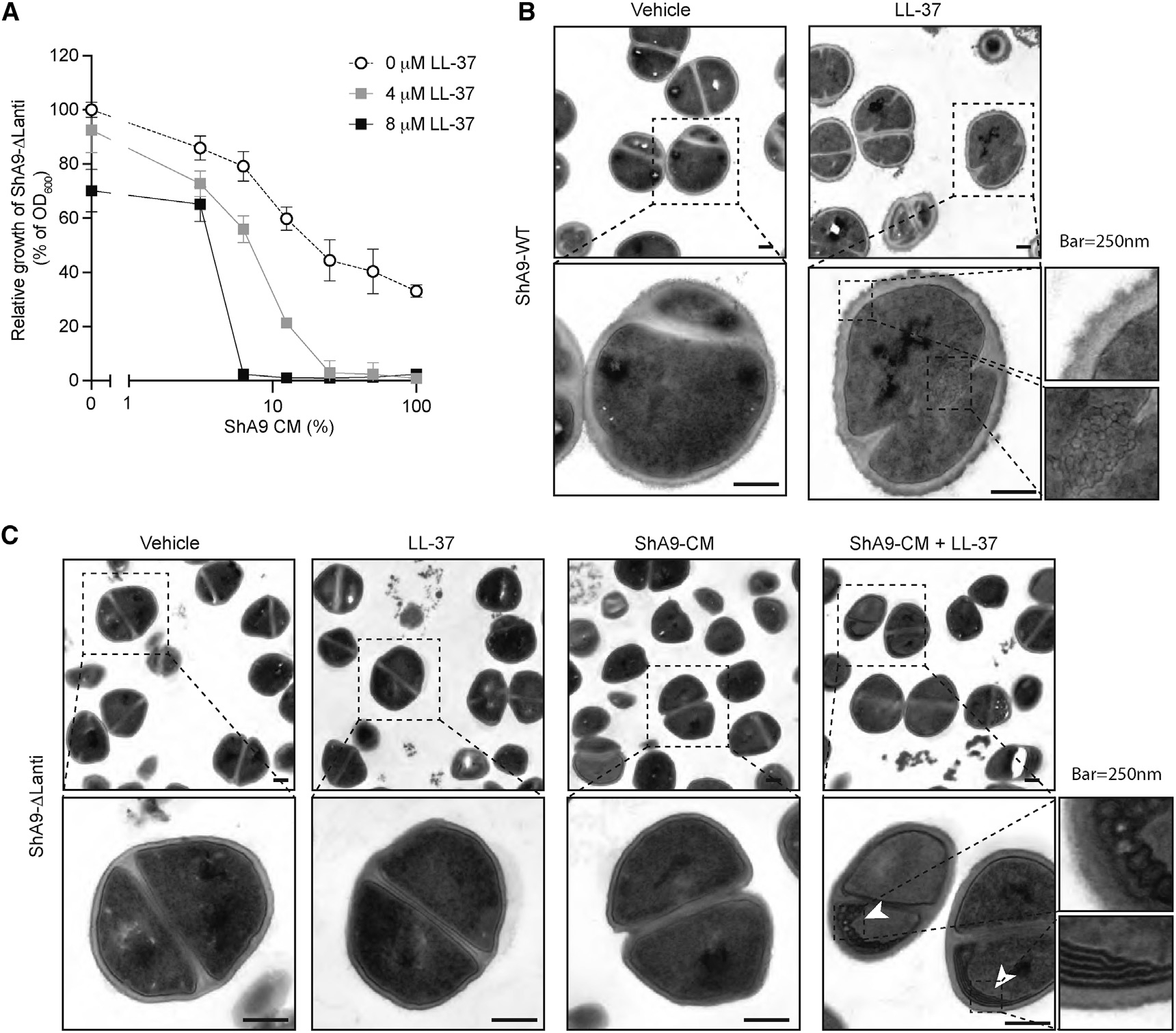
Antimicrobial synergy between antibiotics produced by CoNS and host cathelicidin .(A) Dose-response curves for the antimicrobial activity of conditioned medium (CM) containing ShA9 lantibiotics against ShA9-ΔLanti and in the presence of LL-37. Data represent mean ± SEM of 3 technical replicates. (B and C) Image of electron microscopy of ShA9-WT (B) and ShA9-ΔLanti (C) exposed to LL-37 (4 μM), ShA9 conditioned medium (CM; 25%), or the combination. Scale bar: 250 nm. Image is representative of similar results from indicated biological replicates (n = 3).

To investigate the mechanism by which CoNS strains with antibiotic activity are more sensitive to host AMPs, we incubated ShA9-ΔLanti in the presence of conditioned medium (CM) from ShA9-WT containing its secreted lantibiotics and further added 4 or8 μM of LL-37. The combination of sub-antimicrobial doses of LL-37 with ShA9 CM containing lantibiotics synergistically inhibited growth of ShA9-ΔLanti (Figure 5A). A similar synergy between another lantibiotic and a human AMP was seen with *S. hominis* C6 strain, a strain lacking antimicrobial activity, when LL-37 was combined with Pep-5, a lantibiotic produced by *S. epidermidis* A11 strain (Figure S9). Thus, CoNS strains that lacked endogenous antibiotic activity became more sensitive to human AMPs when the bacterial-derived antibiotics were present.

Electron microscopy of ShA9-WT after exposure to LL-37 (2 μM) showed disruption of cytoplasmic membrane integrity and development of a mesosome-like structure within the cytosol (Figure 5B). The formation of these structures is consistent with the membrane activity of AMPs against *S. aureus*.^29^ In contrast, ShA9-ΔLanti did not show these changes after exposure to LL-37 or ShA9-CM alone but required the addition of a combination of lantibiotic-containing CM and LL-37 to promote membrane damage (Figure 5C). These findings further show the synergistic action of LL-37 and CoNS bacteriocins and suggest they act together to through membrane activity.

### AMPs from skin and antimicrobials from CoNS cooperate to shape the microbial community

**Figure 6.**
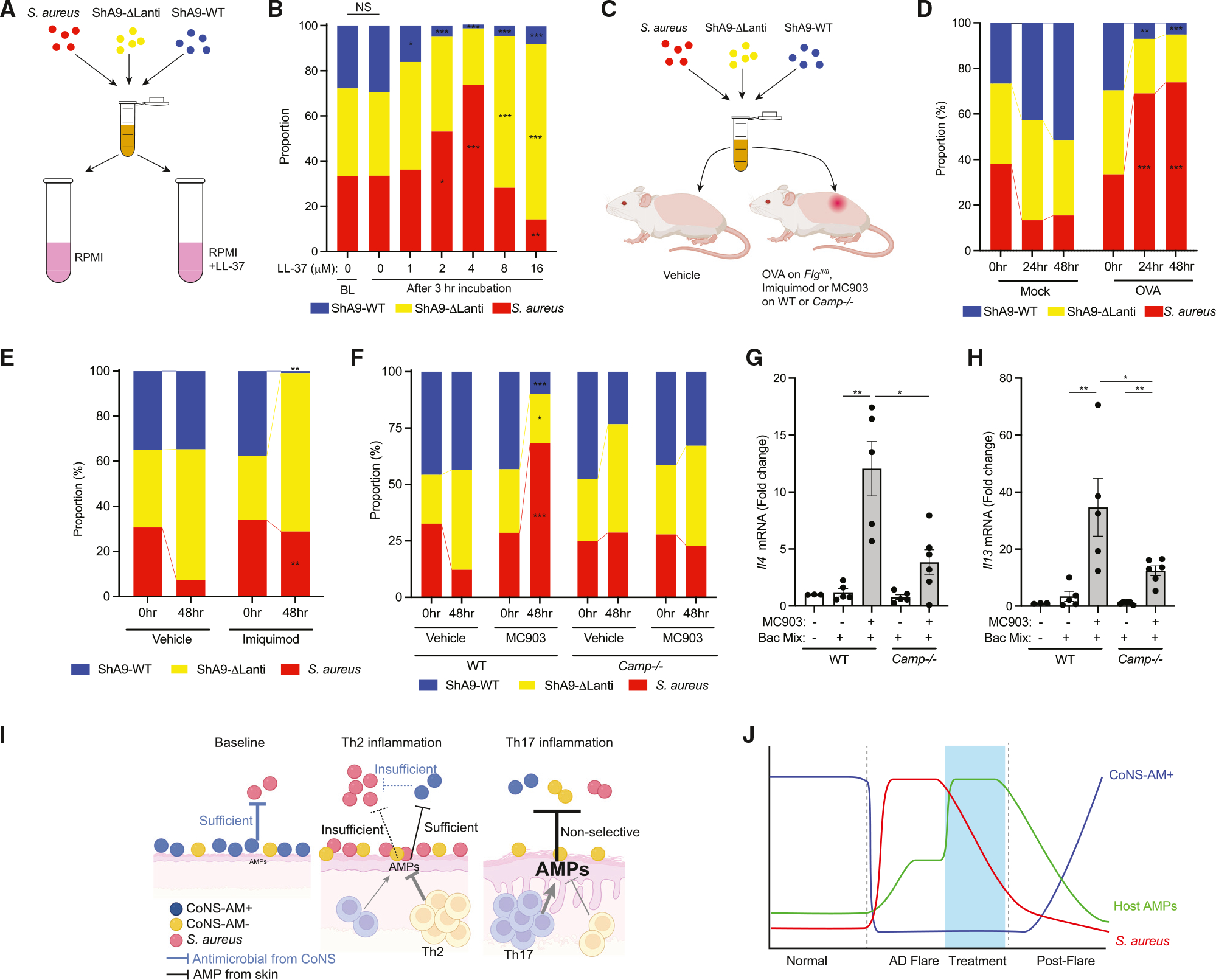
Cathelicidin and antibiotics produced by CoNS cooperatively shape the microbial community during inflammation (A and B) *In vitro* competition among *S. aureus* (ATCC35556), ShA9-WT, and ShA9-ΔLanti mutant in the presence of a low-dose LL-37. Data represent mean of 3 technical replicates. p value (*p < 0.05, **p < 0.01, and ***p < 0.001) was calculated to baseline (BL) by two-tailed unpaired parametric t test. NS, not significant. (C–F) Evaluation of microbial community shifts originally composed of similar CFUs of *S. aureus* (ATCC35556), ShA9-WT, and ShA9-ΔLanti on OVA-sensitized skin of *Flg*^*ft/ft*^ Balb/c mice after 24 and 48 h (D), on imiquimod-treated skin of WT Balb/c mice (E), or on MC903-treated skin of WT or *Camp*^−/−^ Balb/c mice (F) after 48 h. 0 h samples were obtained immediately after bacteria application. Data represent mean of biological replicates from independent mice (D: n = 6, E:n =5 [vehicle] or n = 7 [imiquimod, IMQ], F: n = 5 [WT/vehicle, WT/MC903, *Camp*^−/−^/vehicle] or n = 6 [*Camp*^−/−^/MC903]). p value (*p < 0.05, **p < 0.01, and ***p < 0.001) was calculated by two-tailed unpaired parametric t test (D: vs. mock, E and F: vs. vehicle). (G and H) Expression of *Il4* (G) and *Il13* (H) in the skin treated as in the experiment of (F). Bacterial mixture (Bac Mix) is an equal mixture of *S. aureus*, ShA9-WT, and ShA9-ΔLanti. Each dot represents the average of duplicate technical replicates from an individual animal (n = 3–6 as data shown individually) and data represent mean ± SEM. p value (*p < 0.05 and **p < 0.01) was calculated by two-tailed Mann-Whitney U test. (I) Schematic drawing of antimicrobial defense system in healthy skin, during Th2 inflammation, and during Th17 inflammation. CoNS-AM+, antibiotic-producing CoNS. (J) Fluctuations in *S. aureus*, host AMPs, and CoNS that produce antibiotics during sequential stages of AD.

We next conducted a series of experiments *in vitro* with mixed bacterial cultures and a combination of host AMPs and CoNS-derived antibiotics. This system provided a simplified model of the inflammatory environment in skin by assessing the contribution of both the host and CoNS strains on *S. aureus* survival. A mixture of ShA9-WT, ShA9-ΔLanti, and *S. aureus* was incubated with the human AMP LL-37 at comparable concentrations observed in atopic or psoriatic inflammation^10^ (Figure 6A). When LL-37 was added at relatively lower concentrations (1–4 μM) to the bacterial mixture, a shift in the relative proportion was observed to favor growth of *S. aureus* and ShA9-ΔLanti over the antibiotic-producing ShA9-WT (Figure 6B), thus mimicking the shifts in these bacteria seen in atopic dermatitis. In contrast, addition of higher concentrations (8–16 μM) of LL-37 inhibited both the antibiotic-producing CoNS and *S. aureus*.

To determine if the predicted shifts in the microbial community induced by host AMPs and bacteria with antibiotic activity could be established *in vivo* on mouse skin with Th2 or Th17 inflammation, Balb/c *Flg^ft/ft^* mice were sensitized with OVA or PBS as control, and a mixture composed of similar colony-forming units (CFUs) of ShA9-WT, ShA9-ΔLanti, and *S. aureus* was applied to back skin (Figure 6C). On the mock-treated non-inflamed skin, the proportion of antibiotic-producing CoNS expanded and correlated with a decrease in *S. aureus* at 24 and 48 h compared with the baseline (Figure 6D). On skin with Th2 inflammation, antibiotic-producing CoNS strains were selectively lost, and the proportion of *S. aureus* increased. In contrast, although greater loss of antibiotic-producing CoNS was observed on imiquimod-treated skin compared with OVA-treated skin, *S. aureus* did not increase (Figure 6E). These results paralleled the observations *in vitro* and show that a high level of AMPs induced in Th17 inflammation can inhibit *S. aureus* despite lesser survival of antibiotic-producing CoNS strains. These data are consistent with the relative shifts in microbial abundance observed in psoriasis and atopic dermatitis (Figure 3B and S4).

To test the contribution of *Camp* to the microbial shifts found on inflamed skin, a similar approach was applied to *Camp*^−/−^ or WT mice in which Th2 inflammation was induced by MC903. On the WT skin treated with MC903, a similar shift in absolute abundance of three bacterial strains was observed as on OVA-treated skin: a selective loss of antibiotic-producing CoNS and relative increase in *S. aureus* (Figure 6F). In contrast, on the MC903-treated skin of *Camp*^−/−^ mice, such shifts in the microbial proportion were not observed. In the MC903-treated inflamed skin, greater induction of *Il4* or *Il13* was observed after application of three bacterial mixtures in WT mice than in *Camp*^−/−^ mice (Figures 6G and 6H). These observations suggest that, in the absence of cathelicidin, survival of antibiotic-producing CoNS can partially protect against *S. aureus* and decrease the contribution of this bacteria to exacerbate Th2 inflammation.

## DISCUSSION

Skin with allergic inflammation such as atopic dermatitis shows increased colonization and infection by some *Staphylococcus* species such as *S. aureus* and *S. epidermidis*, but the mechanisms underlying these changes in the bacterial community were not understood. Prior work has shown that expression of cathelicidin and β-defensin AMPs is decreased by the action of Th2 cytokines such as IL-4 and IL-13.^10–12^ However, the skin during Th2 inflammation still has a partial increase in host defense gene expression. This observation presented a paradox, as it could not explain the increase in *S. aureus* growth. In the current work, we now show that the expression of host AMPs during inflammation inhibits survival of beneficial commensals: antibiotic-producing strains of CoNS. Under these conditions of allergic inflammation, both lesser AMPs and lesser antibiotic-producing strains of CoNS enable *S. aureus* to survive. During Th17-dominant inflammation, the higher AMP levels produced by the skin provide sufficient restriction of bacterial growth. On non-inflamed skin, antibiotic-producing strains of CoNS can control *S. aureus* colonization. These results provide a new model for understanding how the skin controls the growth of *S. aureus* and explains why this pathogen successfully colonizes and frequently infects patients with atopic dermatitis (Figure 6I).

Bacteria produce a wide range of antibiotic molecules to inhibit or kill other microbes in their environment. These molecules include bacteriocins such as lantibiotics and other peptides, as well as proteases and a variety of metabolic products.^30^ Mammalian AMPs such as cathelicidins have been reported to synergize with some bacteriocins to enhance their antimicrobial activity.^23,31^ However, we show here that this synergistic activity comes at a cost to the bacterial strains that produce antibiotics. We observed that AMPs induced by the skin under Th2-dominant inflammation cause a selective elimination of antibiotic-producing CoNS strains, shifting the environment to favor *S. aureus* and CoNS strains that do not produce antimicrobial activity. This shift was demonstrated *in vitro* through direct observations of bacterial survival under mixed bacterial culture conditions in the presence of the human cathelicidin LL-37, observed on the skin of patients with atopic dermatitis, and further demonstrated on mice. *Camp* was a significant contributor to the inhibition of both *S. aureus* and antibiotic-producing CoNS since *Camp*^−/−^ mice treated with MC903 did not decrease the survival of ShA9 (an *S. hominis* strain that produces lantibiotics). In these mice lacking *Camp*, the survival of this strain enabled it to inhibit *S. aureus* survival. This was in direct contrast to control mice expressing cathelicidin, where the greater loss of antibiotic-producing CoNS led to increased survival of *S. aureus*. Thus, Th2 skin inflammation drives a partial increase in AMPs that inhibited beneficial CoNS and shifted the overall skin environment to one that favors growth of *S. aureus*.

The findings here show the importance of a dynamic range of antimicrobial molecules on the skin: very low AMPs but abundant antibiotic-producing CoNS are protective on non-inflamed skin, slightly induced AMPs and loss of antibiotic-producing CoNS during Th2-dominant inflammation are the least protective, and the most highly induced host defense gene pattern during Th17 inflammation provides optimal defense against *S. aureus* despite the compromise of the epidermal barrier. Accordingly, skin with atopic dermatitis showed increased bacterial burden and *S. aureus* compared with psoriatic lesional and healthy skin. Interestingly, psoriasis skin also contained an increased proportion of *S. aureus*, but not healthy skin, despite a much higher induction of AMPs in psoriasis than healthy skin. Similar data have also been observed in separate analysis of human skin by shotgun metagenomic analysis.^14,25,32^ Since *S. hominis* lantibiotics exhibit a much stronger and highly selective antimicrobial activity against *S. aureus* than host AMPs like cathelicidin,^23^ we hypothesize that healthy skin is protected (in part) against *S. aureus* by antibiotics produced by commensal bacteria, while psoriatic lesional skin may permit more survival of *S. aureus* due to the loss of these protective organisms. This is supported by observations in the present study of the growth of mixed bacterial communities under controlled conditions *in vitro* and on mice. Additional factors, such as the surface pH, lipids and physical barrier properties also play an important role in shaping the microbiome. Keratinocytes are also a source of additional host defense antimicrobial molecules, including nicotinamide adenine dinucleotide phosphate oxidases that generate reactive oxygen species (ROS), and antimicrobial lipids.^33,34^ Interestingly, skin inflammation associates with accumulation of ROS or a decreased amount of sphingosine, an antimicrobial lipid that acts on *S. aureus*, in the skin.^35–37^ It will be of interest to investigate how alterations of additional host defense molecules influence the dysbiosis of microbial community in various skin inflammatory diseases.

After topical application of MC903 followed by exposure to *S. aureus*, *Il4ra*^−/−^ mice had increased Th17 and decreased Th2 and ILC2 cell populations in the skin compared with WT mice. The application of topical steroids to mice with Th2 inflammation also increased AMP response and, like the *Il4ra*^−/−^ mice, resulted in decreased *S. aureus*. These data in mice are consistent with clinical observations in humans that have reported that the use of immunosuppressive agents or drugs targeting IL-4Rα in patients with atopic dermatitis results in decreased *S. aureus* colonization of inflamed skin.^38,39^ This response during Th2 inflammation is opposite to the increased incidence of microbial infections that occur with use of immunosuppressives in patients without atopic dermatitis.^40,41^ This paradox, why an immunosuppressive treatment could decrease bacterial survival on the skin, is likely explained by the capacity of these drugs to decrease Th2 signaling and shift toward an increased expression of multiple AMPs and relatively higher levels of IL-17A.^20^ Such a response suggests that inhibition of Th2 signaling during allergic inflammation will lead to a peak in host defense that coincides with a decrease in *S. aureus* on the skin (Figure 6J).

In addition to the quantity of AMPs expressed at the epidermis, the individual sensitivity of bacteria to host AMPs is an important factor that determines the shape of the microbial community on the skin surface. Our data show that the production of antibiotics by CoNS can be self-destructive in the presence of host AMPs. Many mammalian AMPs, including cathelicidin and β-defensins, possess common features such as a positive change and amphipathic structures, which allow them to interact with negatively charged bacterial cell membranes, resulting in membrane disruption.^42^ Lantibiotics or α-helical bacteriocins derived from bacteria can kill other bacteria by similar mechanisms.^31,43^ Overall, we conclude that innate immune defense of barrier tissues is mediated by diverse AMPs and possibly even more diverse antibiotics produced by a variety of strains of commensal bacteria.

*Staphylococcus* and other Gram-positive bacteria persist on the skin in part because they have complex AMP resistance mechanisms, including proteolytic degradation, circumvention by alteration of membrane change, and export by efflux pumps.^44^ Multiple peptide resistance factor (*mprF*) is a gene commonly expressed by *Staphylococcus* that confers resistance to both classical AMPs and *S. hominis* lantibiotics through alteration of membrane charge.^28,45^ Thus, the synergistic antimicrobial action between lantibiotic and AMP may be attributed to similar mechanisms of antimicrobial action that overwhelm the resistance mechanisms. Additional studies are necessary to understand the resistance mechanisms within the microbial community in an environment where AMPs, microbiome-derived antibiotics, and other host antimicrobial factors coexist.

The microbial communities that inhabit barrier tissues are diverse, with significant variability between the types of epithelial surfaces and between individuals.^46,47^ However, the composition of these communities has common characteristics that distinguish the individual, tissue, location on the body, and disease.^48^ Specific members of these microbiomes are increasingly thought to be beneficial to the host,^49–51^ while others are more detrimental to health.^52,53^ Therefore, understanding the immune mechanisms by which barrier tissues regulate their microbiome is a critically important area of investigation. The observations shown here *in vitro* and on mice correlated well with findings in humans, the first such demonstration with mixed bacterial communities. However, the ecology of natural microbial communities is much more complex, and the contribution of other organisms in addition to CoNS remains poorly understood. Many questions also remain as to regional differences in survival and the role of early life exposures to establish the commensal community.

In the present study, we focused on the antimicrobial response of epidermal keratinocytes, as this cell type represents the first cells in direct contact with the skin microbiome. However, scRNA-seq in OVA-sensitized mouse skin or human skin with atopic dermatitis has demonstrated that several other resident cell types contribute to Th2 skin inflammation, involving signaling pathways in fibroblasts or cross talk between fibroblasts and dendritic cells.^54–56^ In addition, interaction between *S. aureus* and various myeloid cells, such as mast cells, eosinophils, and basophils, also influences Th2 inflammation.^57–60^ Blocking neutrophil infiltration by CXCR2 blocking antibody improved MC903-induced skin inflammation in mice,^61^ suggesting involvement of this cell type. Furthermore, the differentiation of fibroblasts to preadipocytes and degranulation of mast cells are key events to induce cathelicidin to defend against invasive *S. aureus*.^62–65^ It is of interest to further investigate the influence of IL-4Rα signaling on host defense responses of these cells that reside deeper in the skin to better understand the system.

Use of antibiotic-producing bacteria is currently emerging for treatment of several diseases associated with epithelial dysbiosis in the skin and gut.^24,28,66,67^ This study suggests the benefit of an optimization strategy to stabilize the survival of such beneficial microbes on the skin. Interventions that improve the survival or increase the presence of organisms that produce beneficial products on skin could be used as a mechanism to spare use of broad-spectrum antibiotics as well as immunosuppressive agents. Increased appreciation that immune defense at barrier tissues is a result of the action of the hologenome,^68,69^ gene products of both host cells and microbial inhabitants, will greatly advance the understanding of immune function in both health and disease.

### Limitations of the study

In this study, we evaluated alternative models of inflammation in mice and in humans. In mice, Th2 inflammation was induced by MC903 or OVA, and imiquimod or partial thickness skin wounds were used as representatives of Th17-dominant inflammation. It is recognized that each mouse model is not completely identical to the human disease and is subject to limitations. These models are intended to better inform our understanding of the human skin microbiome. Our direct comparison of atopic dermatitis and psoriasis showed shifts in the microbial communities in human conditions of Th2- and Th17-dominant inflammation that were consistent with observations of these disorders made separately and similar to the mouse models employed here. Therefore, the corroboration of results from alternate mouse models supports conclusions that can translate to humans.

## STAR★METHODS

### RESOURCE AVAILABILITY

#### Lead contact

Further information and requests for recourses, raw data, and reagents should be directed to the lead contact, Richard L. Gallo MD, PhD (rgallo@health.ucsd.edu).

#### Materials availability

All reagents will be made available on request after completion of a materials transfer agreement (MTA).

#### Data and code availability

- The raw data of scRNA-seq for this study have been submitted in open-source web application server. Database: DDBJ sequence Read Archive (www.ddbj.nig.ac.jp), BioProject ID: PRJDB14428, BioSample ID: SAMD00545788~SAMD00545795, accession number: DRA015287.
- This paper does not report original code.
- Any additional information required to reanalyze the data reported in this paper is available from the lead contact upon request.

### EXPERIMENTAL MODEL AND SUBJECT DETAILS

#### Mouse inflammation models

All experiments involving live animals were done with the approval of the Institutional Animal Care and Use Guidelines of the University of California, San Diego (Protocol number: S09074). To investigate influences of distinct type of skin inflammation on the microbial community on the skin surface, we employed Th2 and Th17 inflammation models using mixed sex of *Il4ra*^−*/*−^ Balb/c (Jackson Laboratory, ME), *Camp*^−*/*−^ Balb/c,^4^ *Flg^ft/ft^* Balb/c or WT Balb/c mice (Jackson Laboratory) (6–8 weeks). Mice were housed under a specific pathogen-free condition with 12hr light/12hr dark cycle at 20–22°C and 30–70% humidity. Mice were randomly selected, the back skin was shaved, treated with depilatory cream, rinsed with water, cleaned with alcohol swab twice. To induce Th2 inflammation, MC903 (50μL of 90μM in ethanol) was applied to the back skin of WT, *Il4ra*^−/−^ or *Camp*^−/−^ mice every day for 14 days. Control mice received equal volume of ethanol. Alternatively, a sterile patch (2 × 2cm) with OVA solution (100 μg/100 μL PBS) or PBS was placed on tape-stripped dorsal skin of *Flg^ft/ft^* Balb/c mice (6–8 weeks, mixed sex) and covered with Tegaderm wound dressing film for 8 days (the patch was replaced every 2 days). Mice received 3 times of 8-day OVA exposures by 2-week intervals. Skin was treated with depilatory cream after the first and second cycle. The dorsal skin was disinfected with alcohol swabs twice, applied with a 6-mm TSB agar disc containing 1 × 10^6^ CFU of *S. aureus* (ATCC35556), and covered with Tegaderm wound dressing film for initial 24 h for quantitative bacterial application. To induce Th17 inflammation, 50 μg of 5% imiquimod cream (Perrigo) or control cream (VWR: Cat#56614–414) was applied to back skin of WT or *Camp*^−/−^ Balb/c mice (4 cm^2^) every day for 1 week. For a wound-induced inflammation model, the back skin of WT Balb/c mice was scratched with 18-G needle to cut the epidermal layer.

#### Treatment of mice with a topical steroid

Back skin of Balb/c WT mice (8 weeks, female) were shaved and treated with MC903 as described above. After decontaminating the skin with alcohol swab twice, *S. aureus* (ATCC35556, 1 × 10^6^ CFU) were applied with a TSB agar disc and whole back skin was covered with Tegaderm wound dressing film for 24 h. Tegaderm and the TSB disc were removed, and then 0.1% Triamcinolone (Perrigo) or Vaseline (50μg) were applied twice on back skin at 24 and 32 h after S. aureus were applied. Skin swab and biopsy was ob tained to measure *S. aureus* survival and immune response of the skin, respectively, at 48 h after bacteria application.

#### Human subjects

All experiments involving human subjects were carried out according to the IRB protocols approved by UCSD (Project#071032). Adult subjects with moderate to severe atopic dermatitis or psoriasis, and matched healthy subjects were recruited form the UCSD, San Diego. Demographic data of all human studies are provided in Tables S1–S4. To correct live bacteria or microbial DNA, swab was prewet with sterile saline or TE buffer supplemented with 0.05% Tween 20 and 0.1% Triton X-100, respectively. Collection of live bacteria was done by swabbing lesional skin on the antecubital fossa of patients with atopic dermatitis and on elbow or upper arm of patients with psoriasis, or upper arm of healthy subjects (3 cm^2^) by stroking 20 times with constant pressure. Live bacterial swab was stored immediately in 85% tryptic soy broth and 15% glycerol at −80°C. DNA swab was stored in DNA/RNA shield Lysis tube (ZymoResearch) at −80°C until DNA extraction process.

### METHOD DETAILS

#### Quantification of cytokine and AMP genes in mouse skin

To determine expression of mRNA for *Il4*, *Il13*, *Il17a*, *Tslp*, *Defb4*, *Defb4* or *Camp*, 6mm punch biopsy was obtained from the mouse back skin. Skin biopsy was stored in 1mL of RNAlater solution at −80°C. Total RNA was extracted with PureLink RNA extraction kit (ThermoScientific Fisher) and one μg of total RNA was reversely transcribed with VERSO cDNA synthesis kit (ThermoScientific Fisher). Predeveloped qPCR assay primer sets (IDT oligo, listed in key resources table) were used for analyzing the expressions of these genes. The mRNA expression of each gene was evaluated by qPCR and normalized to GAPDH expression (key resources table).

#### Measurement of bacterial survival on mouse skin

Bacteria were cultured in tryptic soy broth (TSB) at 37°C overnight, washed with PBS, and re-suspended in PBS at 1 × 10^6^ CFU/5μL. To determine a capacity of antibiotic-producing CoNS, non-antibiotic-producing CoNS or *S. aureus* strain to colonize on the inflamed or non-inflamed skin, an 8mm TSB-agar disk containing each bacterial strain (1 × 10^6^ CFU) was applied on the skin after disinfecting the skin with alcohol swabs twice. The entire dorsal skin was then covered with wound dressing film for quantitative bacterial application and to avoid contamination.^18^ Tegaderm and agar disc were removed 24 h after bacterial application and mice were kept additional 24 or 48 h in clean cage. After bacterial application, up to 2 mice with the same bacterial strain were housed. An agar disc without bacteria was used as negative control. The entire skin sheet was rubbed 50 times with nylon foam swab (Puritan) pre-moisturized with PBS to collect bacteria on the skin surface. Swab sample was suspended in 1mL of 85% TSB/15% glycerol and frozen until analysis.

#### Measurement of bacterial survival on pigskin

Fresh-frozen pigskin sheets were obtained from Loretta Tomlin Animal Technologies (Livermore, CA) and sanitized by surgical brush with 3% chloroxylenol. The skin sheet was cut into 2.5 cm × 2.5 cm and rinsed with sterile PBS more than 20 times to remove chloroxylenol residue. Synthetic LL-37 (2.5nanomol/50μL H_2_O) or equal volume of vehicle was applied to the surface of sanitized pig skin. Antibiotic-producing or non-antibiotic-producing strain of CoNS (1 × 10^6^ CFU/10μL PBS) was applied on the pigskin. Antimicrobial activity of CoNS strains against *S. aureus* (ATCC35556) is shown in Figure S5. Pigskin sheet was placed on a 6-well plate and incubated at 30°C for 24 h. The entire skin sheet was rubbed 50 times with nylon foam swab (Puritan) pre-moisturized with PBS to collect bacteria on the skin surface. Swab sample was suspended in 1mL of 85% TSB/15% glycerol and frozen until analysis.

#### Measurement of bacterial survival on human skin

To evaluate survival of antibiotic-producing CoNS strains on lesional and nonlesional skin of adult patients with atopic dermatitis, proportion of antimicrobial CoNS was measured in existing swab samples from previous clinical trial^24^ [registered at ClinicalTrials.gov (NCT03158012)] as described below. An antimicrobial CoNS strain from each patient was formulated at 1×10^7^ colony-forming unit (CFUs)/gram in Cetaphil lotion (Galderma) which was confirmed not to affect bacterial viability. Vehicle cream was formulated with an equal amount of saline only. Two grams of the active lotion with live CoNS, or the vehicle alone, was applied to the ventral surface of the arm including lesional (antecubital fossa) and nonlesional skin (forearm) sites. Application was performed twice a day for 7 days. Skin swabs were obtained at day-0 (baseline), on day-4, day-7 and day-11. Day-4 and Day-7 swab was obtained 4 h after the last application.

#### *Staphylococcus* competition assay *in vitro* or on mouse skin

Three *Staphylococcus* strains, *S. aureus* (ATCC35556), ShA9-WT (antibiotic-producing strain) or ShA9-Dlanti mutant (non-antimicrobial strain) were individually cultured in TSB. Bacterial cells were washed with PBS and re-suspended in fresh PBS. For *in vitro* competition assay, three bacterial strains were suspended in RPMI1640 media (1mL) at 1 × 10^6^ CFU/mL each and LL-37 was added at 0, 1, 2, 4, 8, 16μM. Bacterial mixture were incubated at 30°C for 3 h without shaking. For bacterial competition on mouse skin, mixture of three *Staphylococcus* strains (1 × 10^6^ CFU each in 10μL PBS) was applied on back skin (4cm^2^) of *Flg^ft/ft^* Balb/c mice sensitized by OVA, or WT or *Camp*^−/−^ Balb/c mice pre-treated by imiquimod or MC903 as described above after skin was sanitized by alcohol swab twice. Bacteria on the skin surface were harvested by swab method as described above. Proportion of three *Staphylococcus* strains was determined by CFU counting on mannitol salt agar with egg yolk for total *Staphylococcus* CFU and on Barid-Parker agar for *S. aureus*. Relative proportion of *Staphylococcus* species was determined as following; *S. aureus*: CFUs on Baird-Parker CFU/CFUs on mannitol salt agar, CoNS: CFUs on mannitol salt agar – CFUs on Baird-Parker. Ratio of ShA9-WT to ShA9-DLanti mutant were measured by analyzing 12 colonies of pink color CoNS colonies without halo on mannitol salt agar from each sample with colony PCR using lantibiotic-specific primers (key resources table).

#### Count of bacterial colony forming units

Survival of *S. aureus* and CoNS were measured on Baird-Parker agar for *S. aureus* or mannitol salt agar with egg yolk for total *Staphylococcus* species using easySpiral automatic diluter plater with exponential mode or circle mode and automatic colony counter (Interscience Inc., France). To determine total CoNS survival, CFUs on mannitol salt agar were subtracted by CFUs on Baird-Parker agar. To distinguish ShA9-WT and ShA9-ΔLanti mutant, CoNS colonies with pink color without halo were randomly picked from mannitol salt agar plate and conducted colony PCR with gene specific primers for ShA9-lantibiotic-α (key resources table).

#### Measurement of proportion of antibiotic-producing CoNS strains on human skin

Individual isolated colonies of CoNS from human skin were grown on mannitol-salt agar with egg yolk (pink color without egg yolk reaction), randomly picked and transferred to individual wells containing TSB (400μL) in a 96-well cluster tube. Each plate also contained wells with a non-antimicrobial strain of *S. epidermidis* (*S. epidermidis* 1457) as a negative control, a potent antimicrobial strain of *Staphylococcus hominis* (A9 strain) as positive control, and blank wells without bacteria. CoNS were cultured at 37°C overnight with shaking. Growth was evaluated by OD_600_. Bacteria were removed by centrifugation followed by sterile filtration with a 0.22μm membrane. Sterile conditioned media were mixed with fresh TSB at 1:1. The antimicrobial activity released from each CoNS clone was evaluated by culturing 1 × 10^4^ CFU of *S. aureus* (ATCC35556) in 200μL of diluted conditioned media at 30°C for 24 h. Antimicrobial CoNS strains were defined as those that suppressed *S. aureus* growth to less than 50% (I50) of growth seen in negative controls of *S. epidermidis* 1457 conditioned media. To determine frequency of antimicrobial CoNS, up to 48 CoNS vvvcolonies were analyzed.

#### Single-cell RNA sequencing

An eight mm biopsy was obtained from the non-inflamed skin of mock-treated WT Balb/c female mice or inflamed skin of WT Balb/c or *Il4ra*^−*/*−^ Balb/c female mice treated with *S. aureus* application only, or MC903 for 14 days, followed by *S. aureus* for 48 h. Each biopsy was individually processed into small pieces with surgical scissors followed by digestion in Hanks’ balanced salt solution (Thermo Fisher Scientific, #14175095) supplemented with bovine serum albumin (20 mg/mL), 1× antibiotic-antimycotic (Thermo Fisher Scientific, #15240062), DNase I (50 U/ml; Sigma-Aldrich, #04716728001), Liberase TL (0.1 mg/mL; SigmaAldrich Millipore), 20 mM HEPES (Thermo Fisher Scientific), 2 mM sodium pyruvate (Thermo Fisher Scientific), and collagenase type IV (1 mg/mL; SigmaAldrich Millipore). Tissue dissociation was carried out by a 2-h ice-cold incubation in the digestion buffer followed by 45-min incubation at 37°C shaking at 200 rpm. Enzymatic dissociation was terminated by addition of 5 mM EDTA and 10% FBS. Digests were filtered twice through a 40-μm filter and then incubated in red blood cell lysis buffer followed by removal of dead cell using the MACS Dead Cell Removal Kit (Miltenyi Biotec). Single cell RNA libraries were constructed with 10X Genomics Chromium system (10X Genomics). The 10X Genomics Cell Ranger software pipeline with default parameters was used to perform sample demultiplexing, barcode processing, alignment to the mm10 reference genome, and single-cell gene counting. Data were further filtered, processed, and analyzed using the Seurat R toolkit.^70,71^ Filtering of initial data involved selecting cells with >500 features, >800 UMIs, and <10% mitochondrial genes. Additionally, doublets were removed using the DoubletFinder package with the default settings.^72^ Data were normalized and integrated using the NormalizeData and IntegrateData functions with default parameters. Clusters were identified using the FindNeighbors (50 PCs) and then FindClusters function with a range of resolutions. For each resolution, nonlinear dimensionality reduction and visualization were performed with UMAP^73^ using the RunUMAP function (50 PCs), and marker genes for each cluster were determined using the FindAllMarkers function with min.pct = 0.25. The resolution yielding clusters with the most distinct marker genes was chosen for further analysis (0.1). For lymphocyte and keratinocyte analysis, the data were subsetted based on marker genes, contaminating cells were removed based on expression of marker genes, and the above analysis was repeated. Marker genes for each condition were determined using the FindAllMarkers function with min.pct = 0.25. GO analysis was performed with the top 25 differentially expressed genes using Metscape with default parameters.

#### Immunofluorescence

To detect mouse cathelin related AMP (CRAMP), frozen sections of mouse skin (7μm) were fixed with cold acetone, blocked with Image-iT FX signal enhancer, and incubated with rabbit anti-CRAMP IgG (10 μg/mL) for 2 h, followed by goat anti-rabbit IgG-Alexa 488 conjugate (2 μg/mL) (Invitrogen) for 1 h. Nuclei were counterstained with 4′,6-diamidino-2-phenylindole (DAPI).

#### Assessment of bacterial sensitivity to AMPs

*Staphylococcus* strains collected from normal skin from healthy subjects or lesional skin from AD patients were cultured in TSB overnight. Bacteria were washed with PBS and resuspended to 90% RPMI media and 10% TSB. Bacteria (1 × 10^5^ CFU/mL) were incu bated with or without human AMPs (LL-37, hBD-2, hBD-3) or mouse cathelin-related AMP (CRAMP) in 90% RPMI media and 10% TSB (100 μL) in each well of a 96-well plate at 30°C for 24 h. Bacterial growth was assessed by measuring OD_600_. Minimal inhibitory concentration was defined as the lowest concentration which prevent visible bacterial growth (<5% of OD_600_ value of the con trol well).

#### Synergistic antimicrobial assay between LL-37 and lantibiotics

ShA9 or *S. epidermidis* A11 strains, lantibiotic producing CoNS, were cultured in TSB at 37°C with shake overnight. Bacteria were removed by centrifugation, followed by sterile filtration with 0.22μm membrane. Lantibiotics from each strain was precipitated by adding ammonium sulfate into the sterile conditioned media at 70% saturation. The ammonium sulfate precipitation of conditioned media were dissolved in 90% RPMI1640 supplemented by 10% TSB. ShA9-DLanti mutant or *S. hominis* C6 strain were cultured in TSB at 37°C overnight and washed with PBS. These strain was incubated with conditioned media precipitation (0–100%) along with 0, 4, 8mM of LL-37 in 90% RPMI/10% TSB at 30°C for 24 h. Concentration of conditioned media precipitation added was calculated based on the original volume of sterile conditioned media. Bacterial survival was evaluated by measuring OD600 or counting CFUs.

#### Transmission electron microscopy

To observe the effect of synergistic antimicrobial action between host AMP and lantibiotic on ShA9, ShA9-DLanti mutant were incubated in 90% RPMI media/10% TSB containing LL-37 (4 mM), ammonium sulfate precipitation fraction of conditioned media from ShA9-WT (25% calculated from the original volume of media), or combination at 30°C for 30 min. Bacterial cells were washed with PBS twice and immediately fixed by re-suspending in modified Karnovsky’s fixative for 4 h, followed by 1% osmium tetroxide in 0.1 M cacodylate buffer for 1 h on ice. The cells were stained with 2% uranyl acetate for 1 h on ice, dehydrated in a graded series of ethanol (50–100%), washed with acetone, and then embedded with Durcupan. Sections (60 nm) were post-stained with 2% uranyl acetate for 5 min and Sato’s lead stain for 1 min. Grids were viewed using a JEOL JEM-1400Plus (JEOL, Peabody, MA) transmission electron microscope and photographed using a Gatan OneView 4 K digital camera (Gatan, Pleasanton, CA).

#### Full-length 16S rRNA gene sequence and microbiome analysis

Microbial DNA was extracted with PureLink microbiome DNA extraction kit (Thermo Fisher). The full length 16S rRNA gene was ampli fied using the KAPA2G Robust HotStart ReadyMix PCR Kit (KK5701; KAPA Biosystems, Wilmington, Massachusetts, USA) with the following primers: forward, 5′-TTTCTGTTGGTG CTGATATTGC AGRGTTYGATYMTGGCTCAG-3′ and reverse, 5′-ACTTGCC TGTCGCTCTATCTTCCGGYTACCTTGTTACGACTT-3′. The amplification conditions were as follows: denaturation at 94°C for 2min; 35 cycles of 94°C for 15s, 55°C for 15s, and 68°C for 30s. Barcodes were added to the amplified DNA by a second PCR using a PCR Barcoding Kit (SQK- PBK004; Oxford Nanopore Technologies, Oxford, UK). The conditions for the second PCR: denaturation at 95°C for 3min; 35 cycles of 95°C for 15s, 62°C for 15s, and 72°C for 30s. Successful amplification was verified by electrophoresis using 1.0% agarose gel. Barcoded 16S rRNA gene amplicons were purified using AMPure XP (Beckman Coulter) and quantified. Equal amounts of amplicons were pooled with subsequent sequencing library preparation according to the manufacturer’s instructions. For samples in which DNA concentration was lower than the detection limit of the device, we added the same volume of eluate as other samples pooled in the library construction. The library was sequenced using the Minion flow cell R9.4.1 (Oxford Nanopore Technologies) on a GridION nanopore sequencer.

16S rRNA gene sequencing was performed using forearm swab samples from healthy, atopic dermatitis, and psoriasis patients (n = 24, n = 8 per group) by the Oxford Nanopore MinION platform. Adaptor removal was performed using Porechop v.0.2.4.^74^ Quality trimming was performed by Nanofilt v.1.1.0.^75^ SeqKit v.1.3 was used for summary statistics. Species-level counts were quantified from the preprocessed reads using Emu^76^ with taxonomy assigned using the Emu v3.0.0 database which includes rrnDB v5.6 and NCBI 16S RefSeq references. A prevalence curve was plotted for each phenotype to determine the optimal threshold for filtering low abundance species per group which suggested that each species should be detected in at least 2 samples. The resulting number of species per phenotype were the following: normal (n = 247), atopic dermatitis (n = 53), and psoriasis (n = 208) (Figure S4A). To best reflect the major species present in each cohort, sequence information was filtered prior to analysis toward species represented in at least 2 samples although it was notable that skin from healthy volunteers and subjects with psoriasis had more samples with unique species (Figure S4A). For alpha diversity analysis, we used the Kruskal-Wallis H-test, implemented using the SciPy v.1.9.1 Python package,^77^ to compare richness distributions between phenotypes. For beta diversity analysis, we used Aitchison distance,^78^ with multiplicative replacement following *1/N_features_^2^*,^79^ implemented using the Soothsayer v.2022.8.31 Python package; more specifically, the *PrincipalCoordinatesAnalysis* and *Agglomerative* objects for ordination and hierarchical clustering, respectively.

### QUANTIFICATION AND STATISTICAL ANALYSIS

GraphPad Prism version 9.4.0 (GraphPad) and Excel version 16.63.1 (Microsoft) were used for biostatistical analysis. Variance of the distribution of the data from mouse experiments were analyzed first. When the data were with normal distribution, unpaired or paired parametric t test was used. All data were obtained by duplicate technical replicates from each sample and average value of technical replicates from each mouse or biological replicate is individually shown. When the data were with abnormal distribution, Mann-Whitney U-test was used or parametric t test was conducted after log transformation.

### ADDITIONAL RESOURCES

This study includes reanalyzed data from our previous clinical trial [ClinicalTrials.gov (NCT03158012)]. Description: URL https://clinicaltrials.gov/ct2/show/NCT03158012.

## Supplementary Material

1

## Figures and Tables

**KEY RESOURCES TABLE:** 

REAGENT or RESOURCE	SOURCE	IDENTIFIER

Antibodies		

Rabbit Anti-mouse CRAMP	Nizet et al., 2021^4^	N/A
Goat anti-rabbit IgG(Fab)2-AlexaFluor488	Invitrogen	Cat#: A11070, Lot#: 1040038, RRID: AB_2532697

Bacterial and virus strains		

*Staphylococcus hominis* A9	Nakatsuji et al., 2017^23^	N/A
*Staphylococcus hominis* A9 ΔLanti	Nakatsuji et al., 2017^28^	N/A
*Staphylococcus epidermidis* G9	Nakatsuji et al., 2017^23^	N/A
*Staphylococcus epidermidis* D1	Nakatsuji et al., 2017^23^	N/A
*Staphylococcus hominis* C4	Nakatsuji et al., 2017^23^	N/A
*Staphylococcus hominis* C5	Nakatsuji et al., 2017^23^	N/A
*Staphylococcus hominis* PR-A3	Nakatsuji et al., 2017^23^	N/A
*Staphylococcus epidermidis* N009-G7	Nakatsuji et al., 2017^23^	N/A
*Staphylococcus epidermidis* A11	Nakatsuji et al., 2017^23^	N/A
*Staphylococcus hominis* C2	Nakatsuji et al., 2017^23^	N/A
*Staphylococcus hominis* AMT2-A12	Nakatsuji et al., 2017^23^	N/A
*Staphylococcus hominis* AMT4-D12	Nakatsuji et al., 2017^23^	N/A
*Staphylococcus aureus* ATCC35556	ATCC	Cat#: ATCC35556

Chemicals, peptides, and recombinant proteins		

IMAGE-IT FX signal enhancer	Invitrogen	Cat#: I36933, Lot#: 2020111
5% Imiquimod cream	Perrigo	Cat#: NDC45802-368-00, Lot#: 129235
Contril cream for Imiquimod	VWR	Cat#: 56614-414, Lot#: N/A
MC903	TOCRIS	Cat#: 2700, Batch#: 6A/248251
0.1% Triamcinolone	Perrigo	Cat#: NDC45802-055-35
Vaseline (vehicle control for triamcinolone)	Uniliver	Cat#: 67352423
Hanks’ balanced salt solution	Thermo Fisher Scientific	Cat#: 14175095
Abtibiotic-antimycotic cocktail	Thermo Fisher Scientific	Cat#: 15240062
deoxyribonuclease I	Millipore Sigma	Cat#: 4716728001
Liberase TL	Millipore Sigma	Cat#: 05401020001, Lot#: 56708200
HEPES solution	Thermo Fisher Scientific	Cat#: 15630080
Sodium pyruvate	Thermo Fisher Scientific	Cat#: 11360070
collagenase type IV	Millipore Sigma	Cat#: 11088882001, Lot#:
RNA later	Invitrogen	Cat#: AM7021, Lot#: 01085002
Fetab Bobine Serum	GEMINI Bio Products	Cat#: 900-108, Lot#: A08G001
Ovalubumin	MP Biomedicals	Cat#: 950512, Lot#: 8711K
Tryptic soy broth	Millipore Sigma	Cat#: T8907-1KG, used multiple lots
Bacto agar	BD	Cat#: 214010, Lot#: 1229826, used multiple lots
Bair-Parker agar	BD	Cat#: 276840, Lot#: 1272665
Egg yolk tellurite	BD	Cat#: 212357, Lot#: 2019838
Mannitol salt agar	BD	Cat#: 211407, Lot#: 0315698
Egg yolk emulsion	HiMedia	Cat#: FD045-100MLX1VL, Lot#: 0000530825, used multiple lots
RPMI media	GIBCO	Cat#: 11875-093, Lot#: 2436322
TE buffer	Invitrogen	Cat#: 12090-015, Lot#: 1691859
Tween 20	ThermoFisher	Cat#: 28320, Lot#: VJ307818
Triton X-100	ThermoFisher	Cat#: 28314, Lot#: VJ308263
LL-37	Genemed Synthesis, Inc.	Custom Peptide
human beta-defensin 2	Peptide International	Cat#: ODF-4338-s, Lot#: 671212
human beta-defensin 3	Peptide International	Cat#: PDF-4382-s, Lot#: 660912
CRAMP (GLL-33)	Genemed Synthesis, Inc.	Custom Peptide
LongAmp Hot Start Taq 2X Master Mix	New England BioLabs	Cat#: M0533S
Agencourt AMPure XP beads	Beckman Coulter	Cat#: A63881

Critical commercial assays		

PureLink Microbiome DNA extraction kit	Invitrogen	Cat#: A29789, Lot# 2077129
PureLink RNA extraction kit	Invitrogen	Cat#: 12183025, Lot#: 2346694
MACS dead cell removal kit	Miltenyi Biotec	Cat#: 130-090-101, Lot#:5210411028
PCR Barcoding Kit	Oxford Nanopore Technologies	Cat#: SQK- PBK004
KAPA2G Robust HotStart ReadyMix PCR Kit	KAPA Biosystems	Cat#: KK5701

Deposited data		

Single Cell RNA-seq data	This paper	DDBJ sequence read archive (www.ddbj.nig.ac.jp), accession number: DRA015287

Experimental models: Organisms/strains		

Wild-type Balb/c mice	Jackson Laboratory	Also known as Balb/cj, Strain #:000651, RRID:IMSR_JAX:000651
*Camp^−/−^* Balb/c mice	Nizet et al., 2001^4^	N/A
*Flg^ft/ft^* Balb/c mice	Gifted by Dr. Raif Geha (Nakatsuji et al., 2016^18^)	N/A
*Il4ra^−/−^* Balb/c mice	Jackson Laboratory	Also known as BALB/c-*Il4ratm1Sz/J*, Strain #:003514, RRID:IMSR_JAX:003514

Oligonucleotides		

Primers for *Il4* qPCR	Integrated DNA technologies	Mm.PT.58.7882098
Primers for *Il13* qPCR	Integrated DNA technologies	Mm.PT.58.31366752
Primers for *Il17a* qPCR	Integrated DNA technologies	Mm.PT.58.6531092
Primers for *Tslp* qPCR	Integrated DNA technologies	Mm.PT.58.41321689
Primers/Probe for *Camp* qPCR	Applied Biosystems	Forward: CTTCACCAGCCCGTCCTTC, Reverse: CCAGGACGACACAGCAGTCA, Probe: FAM-CAGAGGATTGTGACTTCA-MGB
Primers for *Defb4* qPCR	Integrated DNA technologies	Mm.PT.58.29993789
Primers for *Defb14* qPCR	Integrated DNA technologies	Mm.PT.58.41499310
Primers/Probe for *Gapdh* qPCR	Applied Biosystems	Forward: CTTAGCACCCCTGGCCAAG, Reverse: TGGTCATGAGTCCTTCCACG, Probe: VIC-CATCCATGACAACTTTGGTA-MGB
Primers for ShA9-lantibiotic-alpha qPCR	Integrated DNA technologies	Forward: GAGGAGCTACACCGACTATTAC, Reverse: CACATCTAGAAGAGCAAGCTAATG

Software and algorithms		

GraphPad Prism	GraphPad	Version 9.4.1

Other		

Swab	Puritan	25-1506 1PF BT BL
Tegaderm wound dressing film	3M	Cat#: 1620

## Highlights

- Skin colonization by *Staphylococcus aureus* exacerbates existing Th2 inflammation
- IL-4Rα suppresses but does not eliminate antimicrobial peptide (AMP) expression
- CoNS that express antibiotics are more sensitive to AMPs than *S. aureus* or other CoNS
- Loss of antimicrobial defense from AMPs and CoNS enables *S. aureus* survival on skin
