# Supplementary material for: Competition between skin antimicrobial peptides and commensal bacteria in type 2 inflammation enables survival of S. aureus
Source: Cell Rep. Author manuscript; Available in PMC 2023 Jun 28. (PMC10303920; doi:10.1016/j.celrep.2023.112494)
Supplement: 1 [file NIHMS1905374-supplement-1.pdf]

Cell Reports, Volume *42*

Supplemental information

Competition between skin antimicrobial peptides and commensal bacteria in type 2 inflammation enables survival of *S. aureus*

Teruaki Nakatsuji, Samantha L. Brinton, Kellen J. Cavagnero, Alan M. O'Neill, Yang Chen, Tatsuya Dokoshi, Anna M. Butcher, Olive C. Osuoji, Faiza Shafiq, Josh L. Espinoza, Christopher L. Dupont, Tissa R. Hata, and Richard L. Gallo

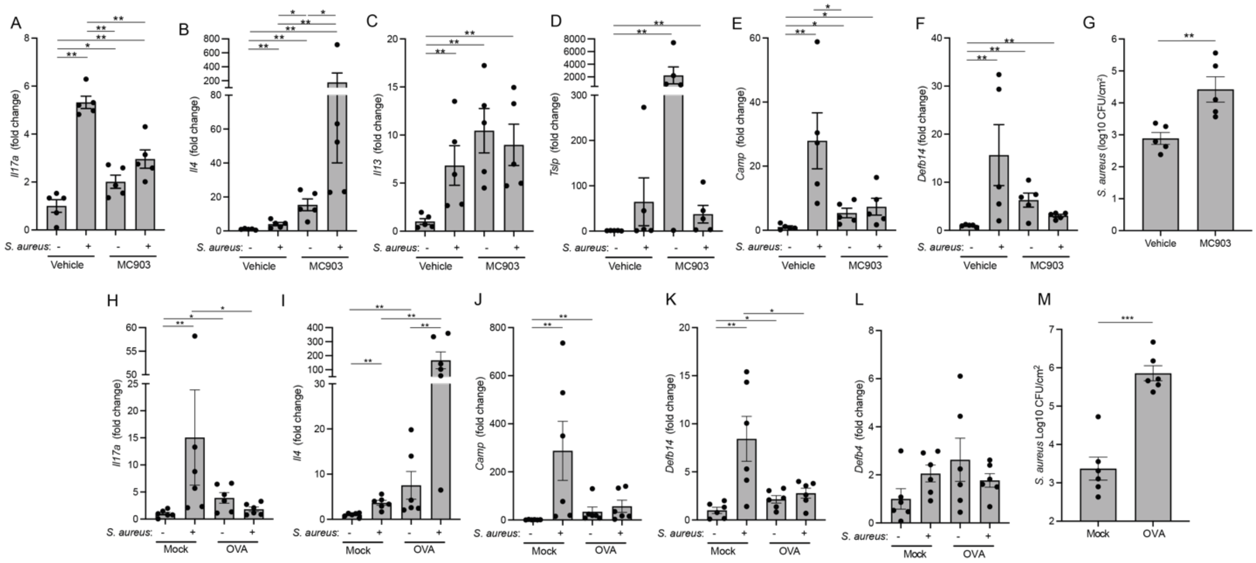

Figure S1. Host defense response to *S. aureus* in the skin of WT mice treated by MC903 or *Flg*$^{ft/ft}$ mice sensitized by OVA.

(A-F) Gene expression for cytokines (A-D) and AMPs (E and F) in the skin of WT Balb/c mice treated with vehicle or MC903 and with or without *S. aureus*. The back skin of WT mice was treated with 50µL of MC903 (90µM in ethanol) or ethanol (vehicle) every 24 hrs for 14 days, and then *S. aureus* (ATCC35556, 1×10^6 CFU) were applied for 48 hours. Data represent mean ± SEM of biological replicates in individual mice (n=5). P-value (*P<0.05 and **P<0.01) was calculated by two-tailed Mann-Whitney U-test or two-tailed unpaired parametric t-test.
(G) Survival of *S. aureus* on the skin of mouse skin treated with MC903 or vehicle for 48 hrs. Data represent mean ± SEM of biological replicates in individual mice (n=5). P-value (**P<0.01) was calculated by two-tailed unpaired parametric t-test.
(H-L) Gene expression for cytokines (H and I) and AMPs (J-L) in the skin of *Flg*$^{ft/ft}$ Balb/c mice with repeated sensitization with PBS (Mock) or ovalbumin (OVA) and with or without *S. aureus*. The back skin of *Flg*$^{ft/ft}$ mice were sensitized by repeated application of 100µg OVA in 100µL PBS or equal volume of PBS for 1 week every other week 3 times and then *S. aureus* (1×10^6 CFU) were applied for 48 hours. Data represent mean ± SEM of biological replicates in individual mice (n=5). P-value (*P<0.05 and **P<0.01) was calculated by two-tailed Mann-Whitney U-test or two-tailed unpaired parametric t-test.
(M) Survival of *S. aureus* on the skin of *Flg*$^{ft/ft}$ mice sensitized by vehicle (Mock) or OVA. Data represent mean ± SEM of biological replicates in individual mouse (n=5). P-value (***P<0.001) was calculated by two-tailed unpaired parametric t-test.

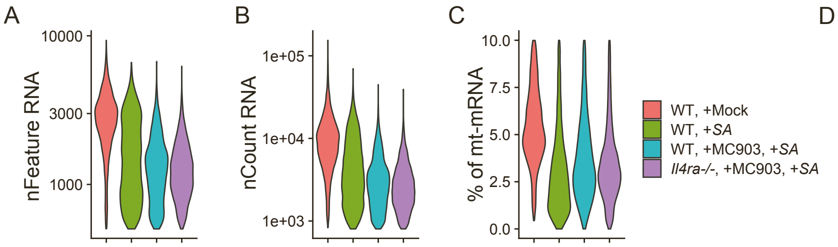

D

Condition	Total cells
WT, +Mock	3957
WT, +*SA*	7781
WT, +MC903, +*SA*	7052
Il4ra-/-, +MC903, +*SA*	8395

E

Cluster	Marker genes	Cell types
0	*Cd3e* -, *Gzmc* +	ILC1/ NK
1	*Cd3e* +, *Foxp3* +	Treg
2	*Cd3e* +, *Il17a* +	Th17
3	*Cd3e* -, *Gata3* +	ILC2
4	*Cd3e* +, *Gata3* +	Th2
5	*Cd3e* +, *Rora* -	Possibly Naïve T cell
6	*Cd3e* -, *Eomes* +, *Gzma* +, *Ifng* +	ILC1/ NK
7	*Cd3e* +, *Mki67* +	Proliferating T cells
8	*Cd3e*+, *Cd8* +	CD8+ T cells

Figure S2. Quality of scRNA-Seq data and identification of lymphocyte clusters in WT or *Il4ra$^{-/-}$* mice treated by *S. aureus* or combination of MC903 and *S. aureus*.

(A-C) Counts of nFeature RNA (A), nCount RNA (B) and proportion of mitochondrial mRNA (mt-mRNA) in scRNA-seq reads.
(D) Total cell counts with high-quality RNA reads after removing cells with low quality RNA reads.
(E) Identification of lymphocyte clusters analyzed by UMAP plot (Figure 1F).
All data in this figure were obtained from 10,000 live cell suspensions pooled equally from five 8 mm punch biopsies of mice independently treated in each group (n=5).

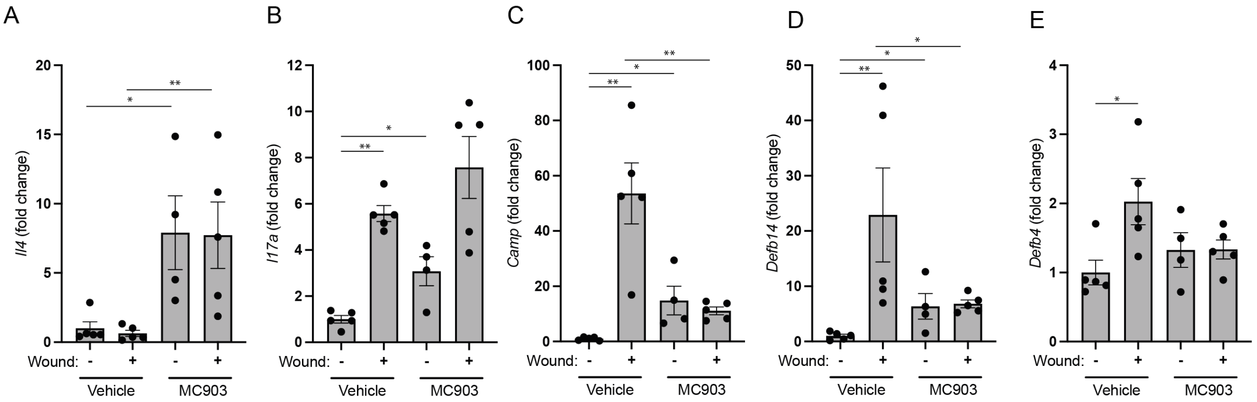

Figure S3. Innate immune response to partial-thickness skin wounds of mice treated by MC903.

(A-E) Gene expression for IL-4 (A) and IL-17A (B) and AMPs (C-E) in the skin of Balb/c mice after wounding by scratching. The back skin of WT mice was treated with MC903 or vehicle for 14 days, and then scratched by an 18 gauge needle. Skin biopsy was taken from the wound edge 48 hours after scratching. Data represent mean ± SEM of biological replicates in individual mouse (n=5). P-value (*P<0.05 and **P<0.01) was calculated by two-tailed unpaired parametric t-test.

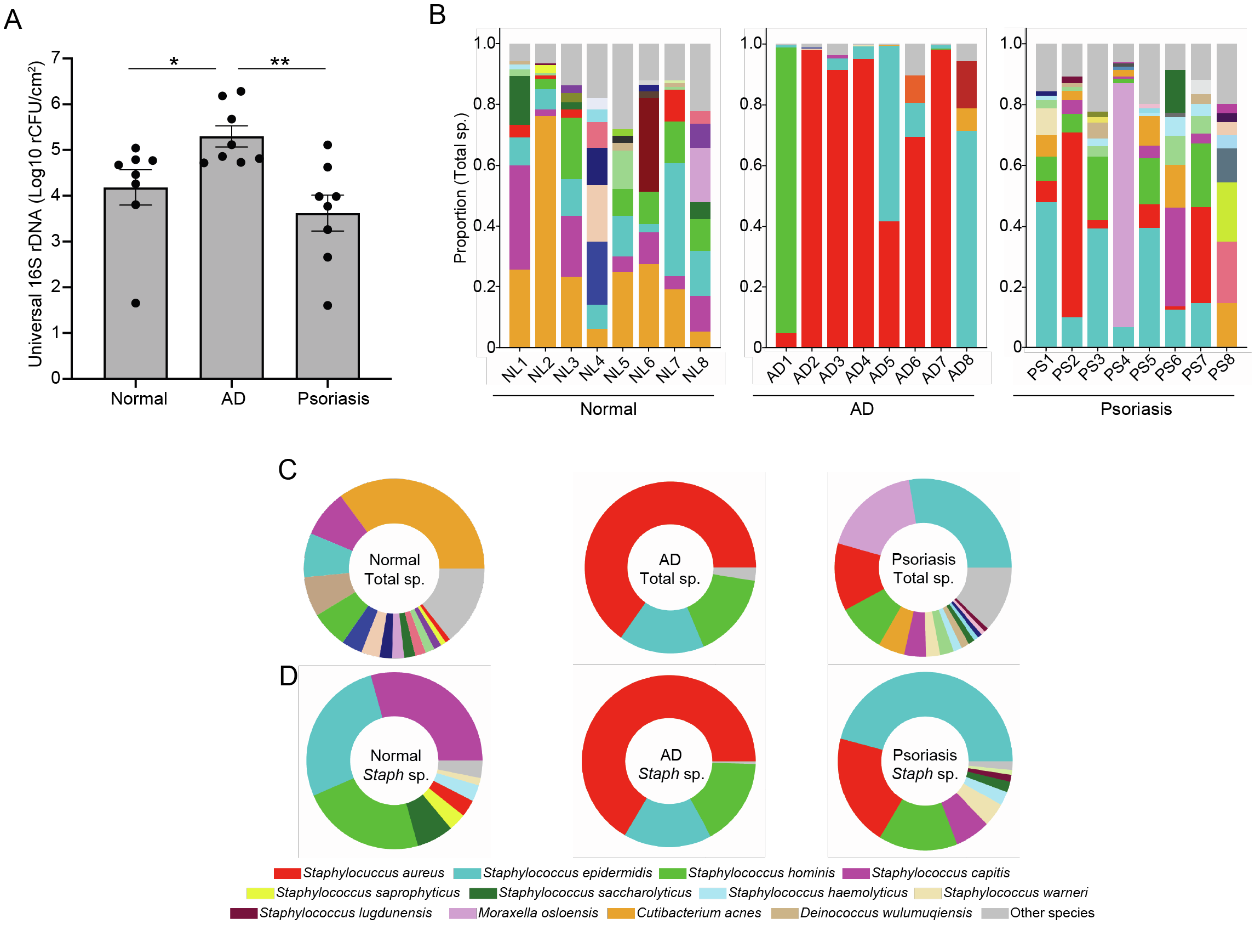

Figure S4. Direct comparison of the human skin microbiome in the normal skin, and skin of patients with atopic dermatitis and psoriasis.

(A) Absolute abundance of skin bacteria as measured by qPCR of 16SrDNA on the arm of healthy subjects and lesional skin on the arm of subjects with atopic dermatitis (AD) or psoriasis. **(B-D)** Long-read 16S rDNA sequencing defines total bacterial species (B,C) or *Staphylococcus* species (D) on the arm of healthy subjects and lesional skin on the arm of subjects with AD or psoriasis. (B) displays individual subjects and (C,D) show mean of data from 8 subjects.

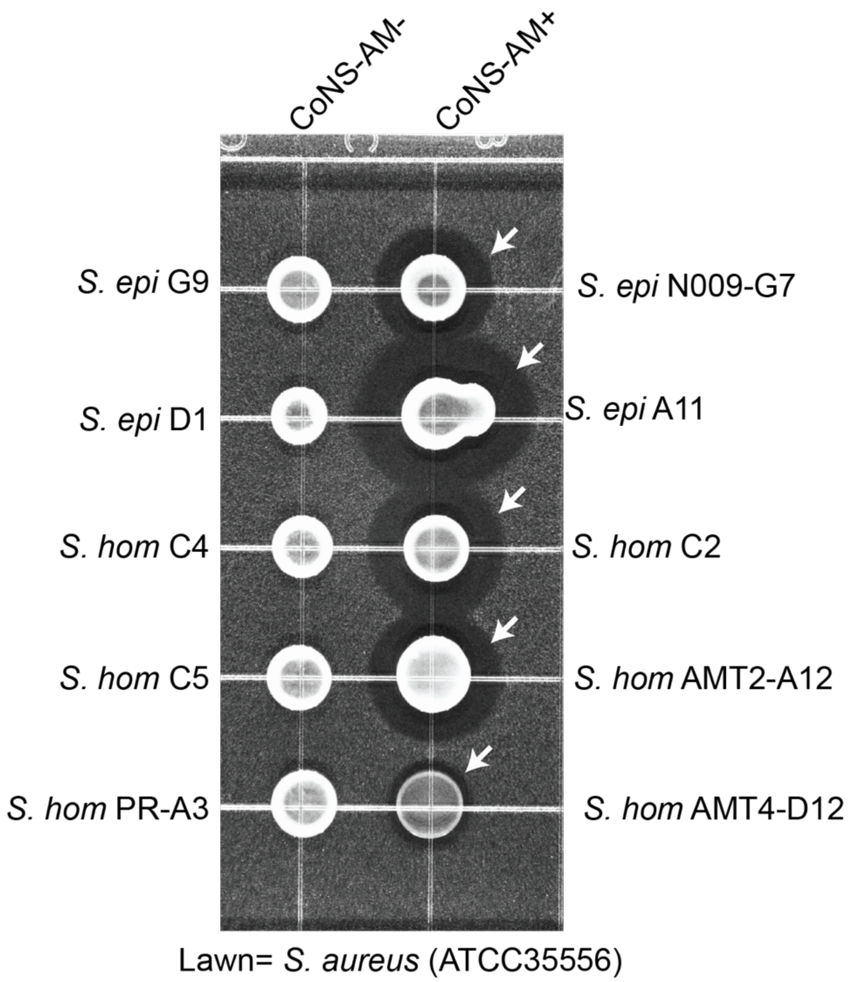

Figure S5. Antimicrobial activity of representative strains of *S. epidermidis* or *S. hominis* with or without capacity to produce bacteriocins against *S. aureus*.

Antimicrobial activity of indicated CoNS-AM+ (with antimicrobial activity) or CoNS-AM- (without antimicrobial activity) strains were tested by radial diffusion assay against *S. aureus* (ATCC35556 known). Arrows indicate zone of growth inhibition of *S. aureus* around tested bacteria. These strains were used for experiments in Figures 3C-3D, 3J and 4F.

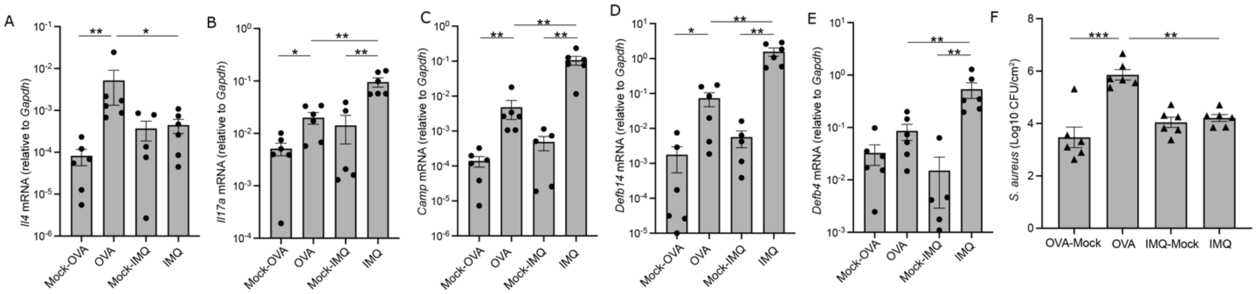

Figure S6. Comparison of gene expression for cytokines and AMPs in the skin of *Flg*$^{ft/ft}$ Balb/c mice treated by OVA or imiquimod.

(A-E) Gene expression for IL-4 (A) and IL-17A (B) and AMPs (C-E) in the skin of *Flg*$^{ft/ft}$ Balb/c mice treated by repeated sensitization with ovalbumin (OVA) or PBS (Mock-OVA) or by repeated daily application of imiquimod (IMQ) or control cream (Mock-IMQ). To induce Th2-predominant inflammation, the back skin of *Flg*$^{ft/ft}$ mice were sensitized by repeated application of 100µg OVA in 100µL PBS or equal volume of PBS for 1 week every other week 3 times. To induce Th17-predominant inflammation, the back skin of *Flg*$^{ft/ft}$ mice were applied with 5% IMQ cream or control cream daily for 7 days.
(F) Survival of *S. aureus* on OVA- or IMQ-treated skin of *Flg*$^{ft/ft}$ mice for 48 hours.
Data represent mean ± SEM of biological replicates in individual mouse (n=6). P-value (*P<0.05, **P<0.01 and ***P<0.001) was calculated by two-tailed Mann-Whitney U-test (A-E) or two-tailed unpaired parametric t-test (F).

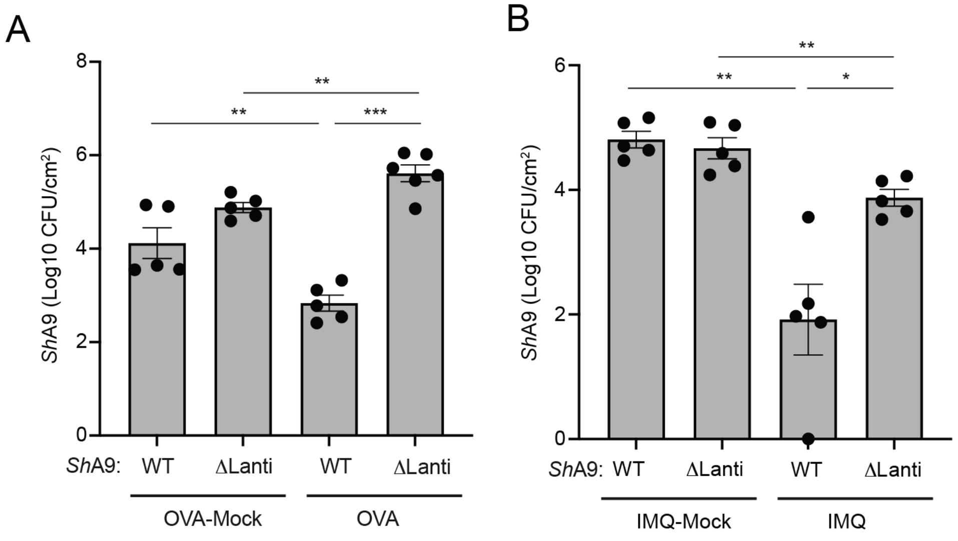

Figure S7. Comparison of survival of ShA9-WT or ShA9-ΔLanti mutant on the OVA-treated skin or imiquimod-treated skin of *Flg$^{ft/ft}$* Balb/c mice.

(A-B) Back skin of *Flg$^{ft/ft}$* mice was sensitized with OVA or PBS (OVA-Mock) to induce Th2 inflammation (A), or treated with 5% imiquimod cream (IMQ) or control cream (IMQ-Mock) to induce Th17 inflammation (B). ShA9-WT or ShA9-ΔLanti mutant was applied on the skin for 48 hours. Data represent mean ± SEM of biological replicates in individual mouse (n=5 or 6 as data from each mice individually shown). P-value (*P<0.05, **P<0.01 and ***P<0.001) was calculated by two-tailed unpaired parametric t-test.

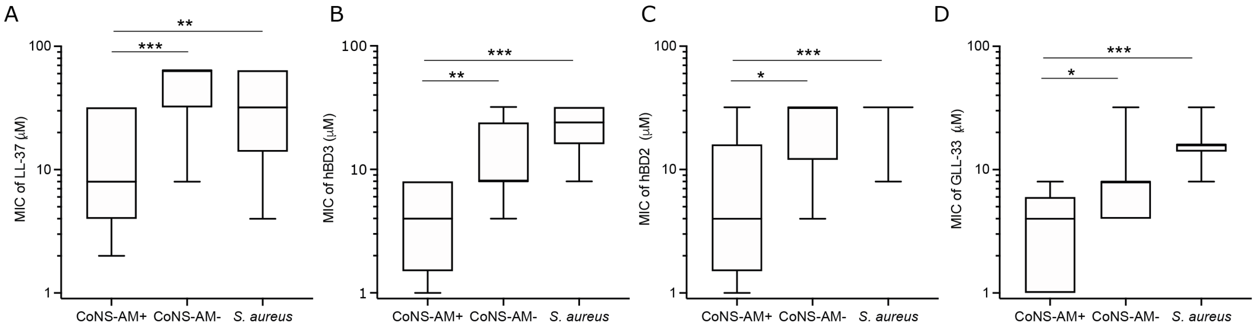

Figure S8. Comparison of Minimal Inhibitory Concentration (MIC) of human or mouse AMPs against CoNS strains with antimicrobial activity, CoNS strains without antimicrobial activity and *S. aureus* strains.

A-D. MIC of LL-37, hBD-3, hBD-2 and GLL-33 to the library of CoNS with antimicrobial activity (CoNS-AM+), CoNS without antimicrobial activity (CoNS-AM-) or *S. aureus* strains. MIC was determined based on the dose-dependent inhibition curve in Figures 4A-4D. Error bar of box and whiskers plot represents mean with range of minimum to maximal value. P-value (*P<0.05, **P<0.01 and ***P<0.001) was calculated by two-tailed Mann-Whitney U-test.

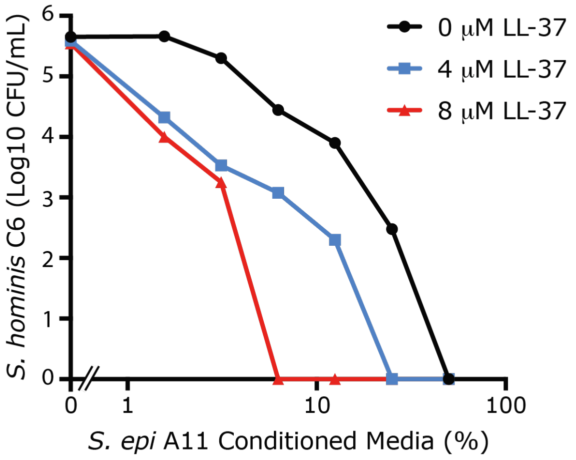

Figure S9. Synergistic antimicrobial activity between pep-5 lantibiotic bacteriocin and LL-37 AMP against *S. hominis* C6, a non-antimicrobial CoNS strain

S. hominis C6 (1×10^3 CFU/mL), a CoNS strain without antimicrobial activity, was cultured in 90% RPMI/ 10% TSB containing 0, 4, 8 mM of LL-37 or ammonium sulfate fraction (70% saturation) of sterile conditioned media from overnight culture of *S. epidermidis* A11, a strain that produce pep-5 lantibiotic bacteriocin, at 30°C for 24 hours. Percent of conditioned media was calculated from original volume of sterile conditioned media Antimicrobial activity was evaluated by measuring colony forming unit (CFU) of bacteria on tryptic soy broth agar after 24 hours.

Table S1. Demographic information of subjects with atopic dermatitis who received autologous application of antibiotic-producing CoNS strains (Figure 3A).

Age (mean ± SD)	28.2 ± 16.3
Gender	
Male [N (%)]	2 (20.0)
Female [N (%)]	3 (80.0)
Race	
Caucasian [N (%)]	1 (20.0)
Asian [N (%)]	4 (80.0)

Table S2. Demographic information of normal subjects, patients with atopic dermatitis or psoriasis for proportion of CoNS isolates with capacity to inhibit *S. aureus* (Figure 3B)

		AD subjects (N= 49)	Normal subjects (N= 30)	Psoriasis subjects (N=8)
Age	Mean±SD	33.4±14.1	33.9±18.2	51.0 ±12.2
Gender [N (%)]	Male	21 (42.9)	17 (56.7)	4 (50.0)
	Female	28 (57.1)	13 (43.3)	4 (50.0)
Race [N (%)]	Caucasian	24 (49)	18 (60)	4 (50.0)
	Asian	16 (32.7)	10 (33.3)	2 (25.0)
	Hispanic	3 (6.1)	2 (6.7)	1 (12.5)
	African-American	4 (8.2)	0	1 (12.5)
	Other	2 (4.1)	0	

Table S3. Demographic information of normal subjects, patients with atopic dermatitis or psoriasis for long-read 16S sequencing (Figures S4)

		AD subjects (N= 8)	Normal subjects (N= 8)	Psoriasis subjects (N=8)
Age	Mean±SD	32.38 ± 16.0	35.8 ± 11.8	51.0 ± 12.2
Gender [N (%)]	Male	4 (50.0)	4 (50.0)	4 (50.0)
	Female	4 (50.0)	4 (50.0)	4 (50.0)
Race [N (%)]	Caucasian	3 (37.5)	4 (50.0)	4 (50.0)
	Asian	3 (37.5)	4 (50.0)	2 (25.0)
	African American			1 (12.5)
	Hispanic			1 (12.5)
	Other	2 (25.0)		

Table S4. Demographic information of normal subjects (Figure 4H)

Age (mean ± SD)	
Gender	
Male [N (%)]	3 (60)
Female [N (%)]	2 (40)
Race	
Caucasian [N (%)]	2 (40)
Asian [N (%)]	2 (40)
Hispanic [N (%)]	1 (20)
